# Optoelectronic Neural Interfaces Based on Quantum Dots

**DOI:** 10.1021/acsami.1c25009

**Published:** 2022-04-28

**Authors:** Mertcan Han, Onuralp Karatum, Sedat Nizamoglu

**Affiliations:** ‡ Department of Electrical and Electronics Engineering, Koç University, Istanbul 34450, Turkey; § Graduate School of Biomedical Science and Engineering, Koç University, Istanbul 34450, Turkey

**Keywords:** quantum dot, nanocrystal, neural stimulation, neural interface, optoelectronics

## Abstract

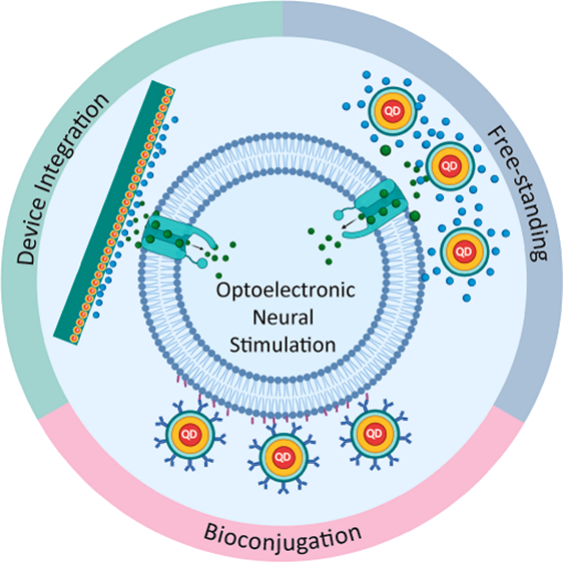

Optoelectronic modulation of neural activity is an emerging field for the investigation of neural circuits and the development of neural therapeutics. Among a wide variety of nanomaterials, colloidal quantum dots provide unique optoelectronic features for neural interfaces such as sensitive tuning of electron and hole energy levels via the quantum confinement effect, controlling the carrier localization via band alignment, and engineering the surface by shell growth and ligand engineering. Even though colloidal quantum dots have been frontier nanomaterials for solar energy harvesting and lighting, their application to optoelectronic neural interfaces has remained below their significant potential. However, this potential has recently gained attention with the rise of bioelectronic medicine. In this review, we unravel the fundamentals of quantum-dot-based optoelectronic biointerfaces and discuss their neuromodulation mechanisms starting from the quantum dot level up to electrode–electrolyte interactions and stimulation of neurons with their physiological pathways. We conclude the review by proposing new strategies and possible perspectives toward nanodevices for the optoelectronic stimulation of neural tissue by utilizing the exceptional nanoscale properties of colloidal quantum dots.

## 1 Introduction

**Figure 1:**
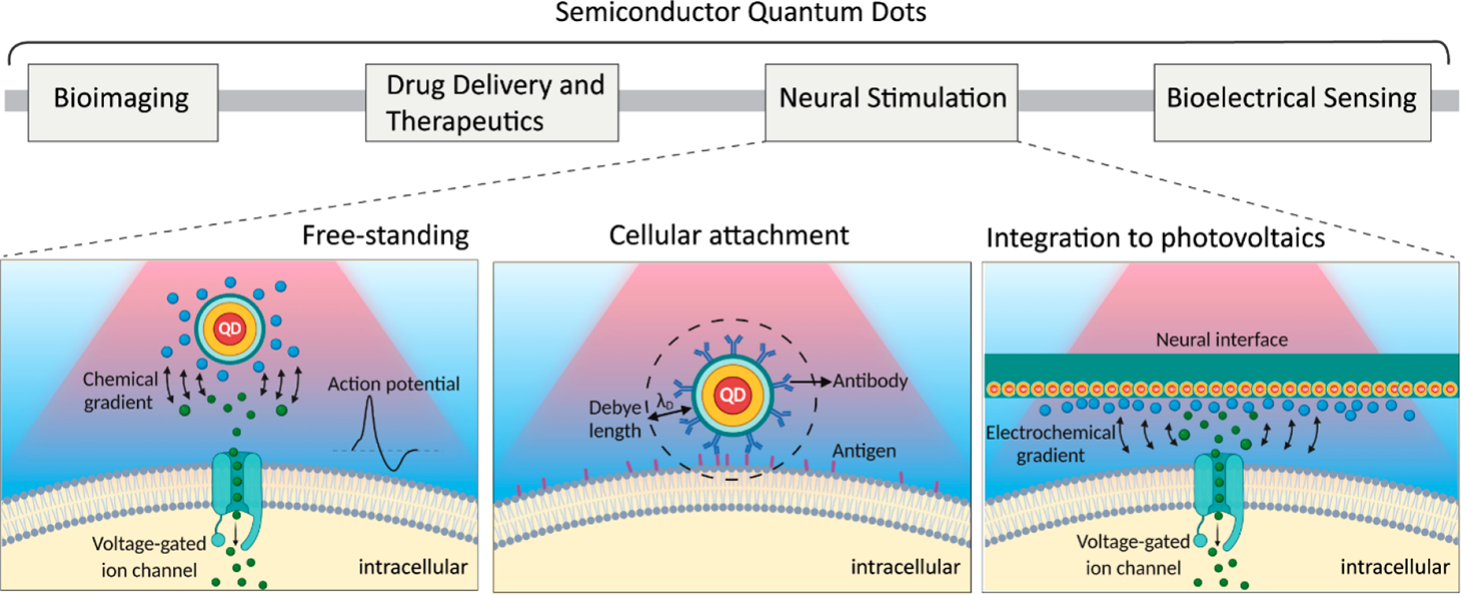
Applications of semiconductor quantum dots for neurotechnology (top). Schematics for the three main configurations that can lead to neural stimulation using quantum dots (bottom). The free-standing configuration represents the interaction between the targeted cells and the QDs in the extracellular medium without any physical, chemical, or biological attachment to the cell membrane. The second configuration (bottom middle) exhibits the interaction between the targeted cells and the QDs, which may bind to the cell membrane through QD–antibody conjugates or via conjugation with target specific ligands, such as peptides and proteins. The third configuration (bottom right) utilizes QDs in thin-film or blend form. Neuron–QD interaction depends on the chemical, physical, or ionic stimuli generated by QDs.

Neural interfaces offer modulation of cellular signals to understand complex neural circuits and treat various disorders including cardiac problems,^1^ paralysis,^2^ epilepsy,^3^ Parkinson’s disease,^4^ and other neurological disorders.^5−7^ The advent of nanotechnology enabled ultrasmall building blocks for neural interfaces with advanced functions that can simultaneously enable efficient, injectable, biocompatible, capacitive, soft and flexible neural interfaces, which can overcome the limitations of their bulky counterparts. Among a wide variety of nanomaterials, colloidal quantum dots (QDs) have exceptional properties such as comparable size with the cell membrane (i.e., 8–10 nm),^8^ ultrasensitive tunability of electronic energy levels via the quantum confinement effect (i.e., size effect), and near-unity quantum efficiency.^9,10^ Because of these favorable optoelectronic properties, QDs have been widely used in a wide range of optoelectronic devices such as light-emitting diodes (LEDs),^11^ photodiodes,^12,13^ solar cells,^14,15^ and phototransistors.^16^ More interestingly, they can be conjugated with a wide variety of biomolecules targeting membrane proteins/receptors with QD–antibody or QD–ligand conjugates for biolabeling,^17,18^ bioimaging,^19−21^ targeted drug delivery or cancer treatment,^21^ biosensing,^22,23^ and neural stimulation (Figure 1). Therefore, colloidal quantum dots hold high promise for future bioelectronic medicine for neurological diseases.

Toward the cellular stimulation goal, QDs operate with the fundamental mechanism of transduction of light to controlled ionic currents for optical control of neurons. Optogenetics, the frontier method for light-triggered control of neural circuits, induces nanoscale photosensitive ion channels in the membrane by permanent genetic modification of the natural structure of the membrane by using a viral vector. However, gene delivery and manipulation methods require a high level of refinement to adapt them for gene-specific conditions^24^ and there are also ethical concerns on the safety of gene therapy for clinical practice.^25^ As a nongenetic approach, today silicon^26,27^ and semiconductor polymers^28^ offer effective optoelectronic material options to control light-triggered modulation of neurons in vitro and in vivo,^29^ which showed promise in cellular stimulation and recovery of vision against blindness at the clinical level.^30^ However, the low absorption coefficient of silicon (383 cm^–1^ at 880 nm)^31^ necessitates the formation of substrates for neural interfaces that are tens of micrometers thick, resulting in rigid devices with high Young’s modulus values in the megapascal and gigapascal range. The mechanical mismatch between the biological tissue and rigid biointerfaces may lead to scar tissue formation as well as the foreign body response, which reduces device performance and functional lifetime for these devices.^32^ On the other hand, quantum dots, which have absorption coefficients that are orders of magnitude higher than that of silicon, can enable ultrathin and flexible devices on silk, poly(ethylene terephthalate) (PET), polydimethylsiloxane (PDMS), and parylene and can be potentially integrated with low Young’s modulus conductive materials such as poly(3,4-ethylenedioxythiophene) polystyrenesulfonate PEDOT:PSS and its hydrogel for tissue-like interfacing with neurons.^33,34^ Furthermore, they may even operate at a single colloid level for the control of neural activity. QDs are also recognized for their outstanding optical stability, showing very little photobleaching or chemical degradation compared to organic dyes.^35^ Approved by the millions of units sold QLED TVs, they can be synthesized at large scales with low cost and combined with solution-processable fabrication techniques that can pave the way toward a widely usable and economically feasible neural prostheses. Therefore, these features make QDs a promising alternative for optoelectronic neural interfaces.

This review discusses the fundamentals and potential of QD-based optoelectronic biointerfaces (Figure 1) converting optical energy to ionic electrical currents to modulate cellular processes, particularly to stimulate neurons. First, the physical mechanisms of QD integrated neural interfaces and dominant biophysical mechanisms of optoelectronic stimulation are discussed. Next, we summarize pioneering studies as well as recent advances for several types of QD optoelectronic neural interfaces while discussing the biocompatibility of such devices. Finally, we discuss future perspectives and new opportunities for future QD integrated optoelectronic biointerfaces. Different from the previous reviews,^26,36−45^ we focus here on state-of-the-art applications for neural interfaces using quantum dots.

## 2 Properties and Neuro-interfacing Configurations of Quantum Dots

**Figure 2:**
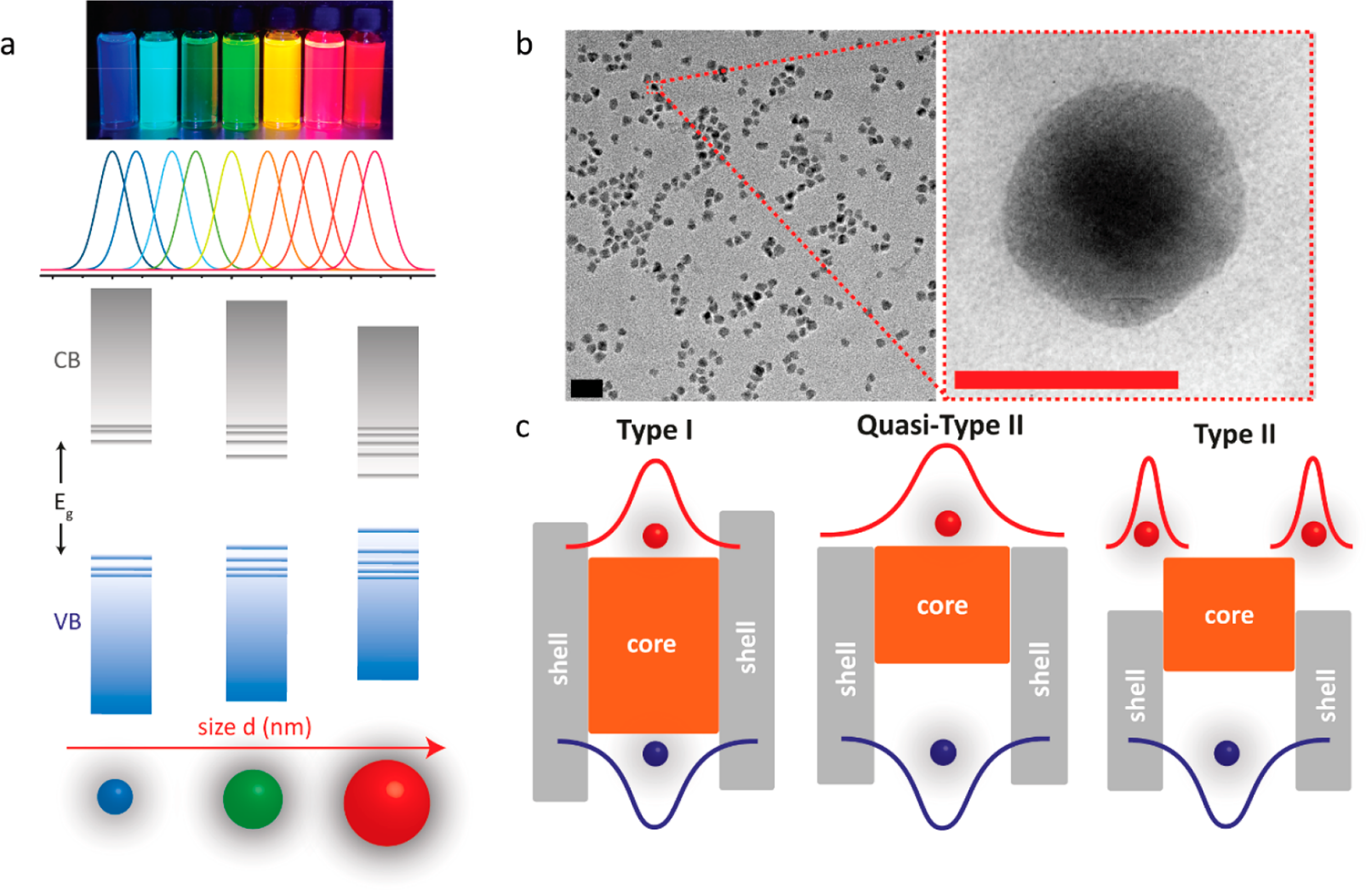
(a) Quantum dots with stepping emission from blue to red (top). Representative photoluminescence spectrum for different size quantum dots (middle). Representative conduction and valence band diagram for different sizes of semiconductor quantum dots (bottom). (b) Representative TEM images for core/shell quantum dots (scale bars: 20 and 5 nm, respectively). (c) Core/shell semiconductor nanoparticle systems with type I, quasi-type II, and type II band alignment.

The interest in quantum dots began with the discovery of quantum size effects in the semiconductor nanocrystals (NC). The synthesis of the first quantum dots in a dielectric glass matrix was followed by their colloidal synthesis in a liquid medium. Moreover, the theoretical studies aimed to model and understand the charge carrier behavior in quantum-confined crystal structures.^46−51^ Now, it is well-known that the squeezing of excitons in quantum dots leads to size-dependent electronic and optical properties (Figure 2a), which makes them attractive materials for various applications such as light-emitting diodes, lasers, solar cells, luminescent solar concentrators, biomarkers, and biolabels.^17,52−54^

Optoelectronic properties of quantum dots were progressively improved via advancements in the field of nanochemistry. Novel synthesis methods resulted in “high-quality” nanocrystals that have near-unity photoluminescence quantum yield (PLQY), narrow emission line widths, sharp absorption profiles, and atomic-level size tunability.^55−57^ For that, production of core/shell nanostructures (Figure 2b), where a small bandgap core nanocrystal is covered with a larger bandgap shell material,^58^ is an effective approach to engineer electronic and optical properties of QDs. Shell growth renders superior surface properties to QDs because of the passivation of surface trap states and tunable energy levels, leading to enhanced optoelectronic characteristics and optical and chemical stability.^58,59^

Depending on the electronic energy level alignment between the core and shell materials in a core/shell QD nanostructure, QDs are categorized into different types (Figure 2c). In type I QDs (e.g., CdSe/CdS, CdSe/ZnS QDs), shell material has a higher conduction band and lower valence band energies compared to the core material, which results in the confinement of electrons and holes in the core with high exciton binding energies. On the contrary, in type II QDs (e.g., CdS/ZnSe, CdTe/CdSe, InP/ZnO QDs), one type of charge carrier localizes in the core, whereas the other charge carrier moves to the shell because of the favorable conduction or valence band energy level of the shell. Moreover, the quasi-type II QD band structure exhibits only partial delocalization of one charge carrier to the shell (Figure 2c). Compared to type I QDs, the exciton binding energies of type II QDs are lower because of the increased physical distance that causes reduced Coulombic force between the bound electrons and holes.^60^ Besides, the increased distance between electron and hole leads to reduced radiative recombination rates and increased recombination lifetimes in type II or quasi-type II QDs.^61^ The increased fluorescence lifetime enables the detection of time-gated signals to monitor cellular behavior.^20^ Likewise, the increase in recombination lifetime is beneficial for charge carrier interactions and transfer to the neighboring materials.

One notable recently emerged application of QDs is neural interfaces. Being efficient absorbers in the visible to near-IR spectrum, QDs are promising materials for photoactive neural interfaces.^62^ QDs can be functionalized to couple them directly with the cell membrane within only nanometer-scale distances using certain antibodies or peptides.^20,63^ For example, avidin-conjugated QDs have been utilized for cell labeling and imaging by attaching through the biotin, the affinity pair of avidin.^64,65^ Moreover, the excitation of QDs can potentially alter the membrane potential due to the electric field generated by the electron–hole separation in the excited QDs. For QDs, there are three possible configurations for cellular stimulation (Figure 1 bottom): (i) free-standing interaction in the extracellular medium with cells, (ii) direct interaction with cellular attachment using surface functionalization, and (iii) integration of QDs into photovoltaic devices either as the photoactive material or electron/hole transport layer. Stimulation of neurons via free-standing configuration has not been achieved yet, possibly because of the strong decay of the electric field generated by the QDs. Because the extracellular medium hosts polarized ions and mobile charge carriers, the screening effect can dampen the generated electric field. Moreover, because the voltage-gated ion channels require at least a 5–10 mV potential difference for switching,^40^ the effective stimulation distance is even shorter than the effective electric field volume. Therefore, the first configuration requires high concentration of QDs that can allow high number of QDs to be near cells. However, the possible cytotoxicity of the concentration effect needs to be considered. The second configuration is convenient to specifically bind QDs to targeted cells without increasing the loading concentration in the extracellular medium to overcome this problem. The advances in bioconjugation schemes and QD surface functionalization have already been proven for bioimaging and targeted treatments using peptide-coated, avidin-coated, and many other techniques.^20^ The same Debye length limitation also applies for the second configuration, but close-proximity operation can potentially enable stimulation of neurons while having acceptable QD on the membrane densities.^22,66^

**Figure 3:**
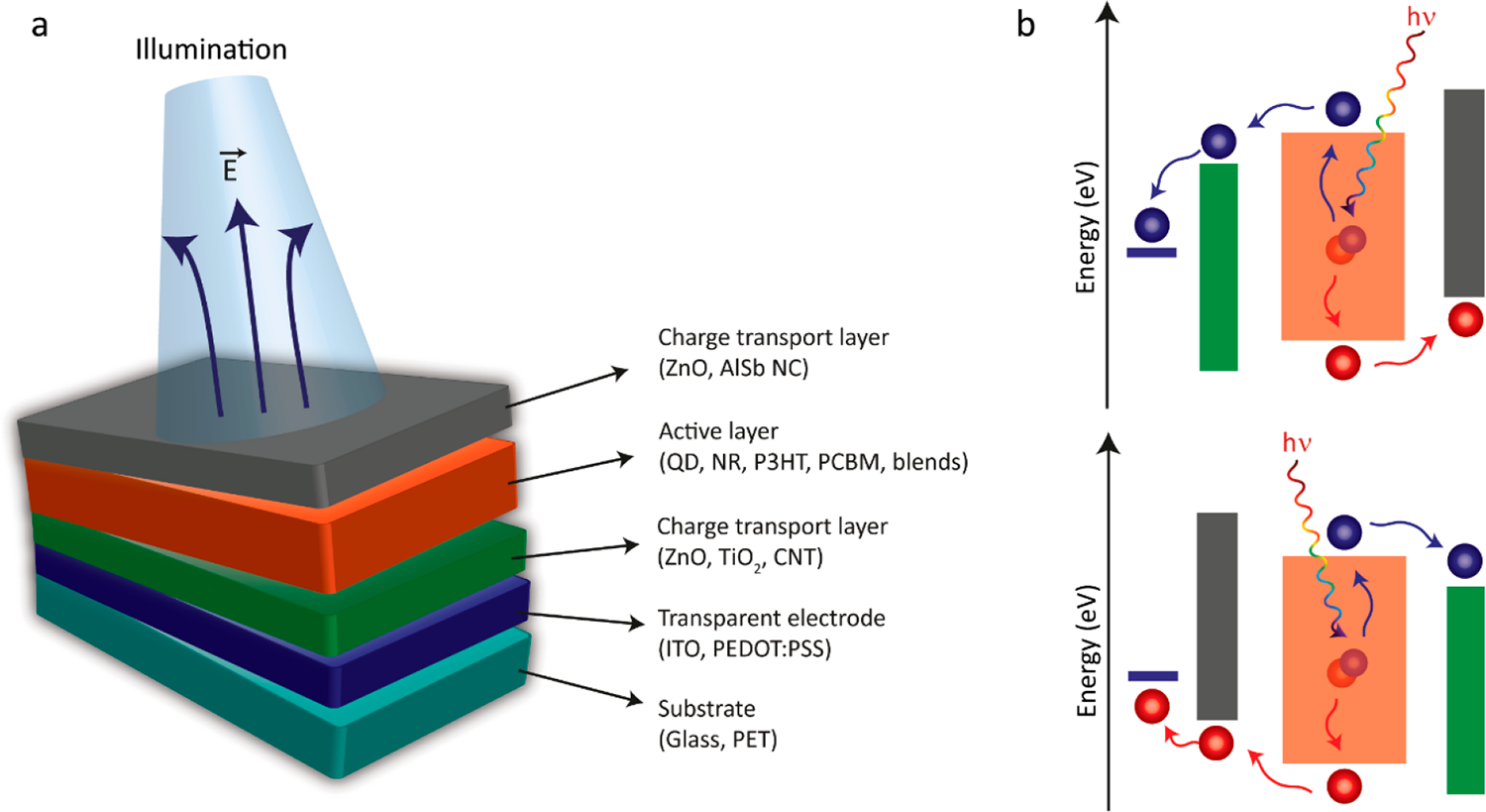
(a) Schematic diagram of a generic photovoltaic biointerface architecture. (b) Energy band diagram of a regular (top) and inverted optoelectronic system (bottom). The layers represent the corresponding layers in panel a. The exciton generation occurs in the active layer upon illumination. Dissociated electron and the hole move toward the charge transport layers according to the energy levels between the layers.

On the other hand, layered photovoltaic architectures with QDs, such as in Figure 3, have exhibited more convenient fabrication procedures and promising results in comparison with the other configurations. Solid films of QDs can effectively convert light energy into ion-based electrical current (photocurrent) in electrolytes, which can achieve extracellular stimulation of nearby neurons. Indeed, this photoelectrical stimulation route using QD films was proven to be effective with few early pioneering studies.^67,68^ Later, inspired from QD-based solar cell devices combined with a bioelectrical perspective, the ability to integrate QD films into various device architectures (e.g., combining QDs with electron and/or hole transport materials, heterojunctions of QDs with semiconducting organic polymers) while considering the device-electrolyte interactions led to advanced quantum dot opto-bioelectronic devices for the optical control of neurons.^69−71^

## 3 Physical and Physiological Mechanisms of Neural Interfaces

### 3.1 Physical Mechanism, Device Design, and Operation Principles

The first process occurring during the operation of QD-based optoelectronic biointerfaces is photon absorption. The absorption of impinging light is dependent on the energy of the incoming photons and bandgap of the QDs in the device structure that can be controlled by the size of the QD. Because of the size-tunability of QDs, highly tunable absorption edges ranging from visible spectrum (e.g., by using CdSe, InP) up to near-IR (e.g., by using PbS) can be built. QDs can manifest linear or nonlinear absorption characteristics by single photon or multiphoton absorption processes, respectively.^72^ This can affect the dependency of the photoresponse of the biointerfaces to the illumination intensity. Biointerfaces operating via single photon absorption demonstrate linear light intensity-photocurrent relationship, whereas multiphoton absorption would result in nonlinear (quadratic or higher) dependency of photocurrent to light intensity.^67^

Upon absorption, electron–hole pairs are created in the QDs. These charge pairs are in a bound state with a binding energy due to the Coulombic attraction between them. QDs exhibit rather large exciton binding energies compared to the thermal energy at room temperature.^61^ One strategy to stimulate neurons is to use the dipole electric field created by the bound excitons.^73^ If neurons are placed sufficiently close to the QDs, the electric field produced by the photogenerated bound excitons can alter the transmembrane potential and evoke action potentials if the induced affect is large enough. Indeed, it was theoretically shown by Winter et al.^73^ that the dipole strengths around the reported values of 30 D for QDs^74^ can generate electric potentials greater than 15 mV at a distance of 10 nm in deionized water, which would be sufficient for the opening of voltage gated ion channels, leading to action potential firing.^75^ However, in an ionic medium similar to the extracellular environment of neurons, the distance for observing the same effect reduces to approximately ∼2 nm due to electric field screening in an ionic medium.^73^ The maximum distance even decreases below that due to the additional structures on the cell membrane (e.g., antibodies) and QDs (e.g., ligands), which may induce further screening effects.^68^ It must be noted that these numbers correspond to the effect of a single nanocrystal. In case of a favorable alignment of many nanocrystals, the collective impact of QDs can be much more pronounced. Yet, despite achieving the coupling of QDs as close as 3 nm to the cell membrane,^63^ photoexcitation of neurons with this strategy was still not achieved.

QD films in photovoltaic biointerface architectures provide a versatile platform for photostimulation. QD films have been widely used in photovoltaic systems as photoactive layer for converting light into electron–hole pairs and photon downshifting layer for converting higher energy photons into lower energy photons.^76,77^ Tight packing of nanocrystals leads to increased charge transportation rates between QDs due to the decreased interparticle distance and thus higher conductivity.^61,78^ Their charge transport properties can be further enhanced via ligand exchange and thermal treatments.^79,80^ In general, the studies demonstrating the successful photoexcitation of neurons have utilized QD solid films that are either coated on a transparent electrode (e.g., ITO) or together with charge transport layers (Figure 3a).^67−69^ Because of the band alignment in device architecture, the photogenerated charges are dissociated (Figure 3b), and one type of charge is accumulated on the biointerface-electrolyte interface where neurons are positioned. The charge accumulation on the interface generates photocurrent in the electrolyte via capacitive and/or faradaic processes, which then depolarizes or hyperpolarizes the neural membrane potential depending on the direction of the photocurrent and the type of the ion channels that are activated.^67−69^

**Figure 4:**
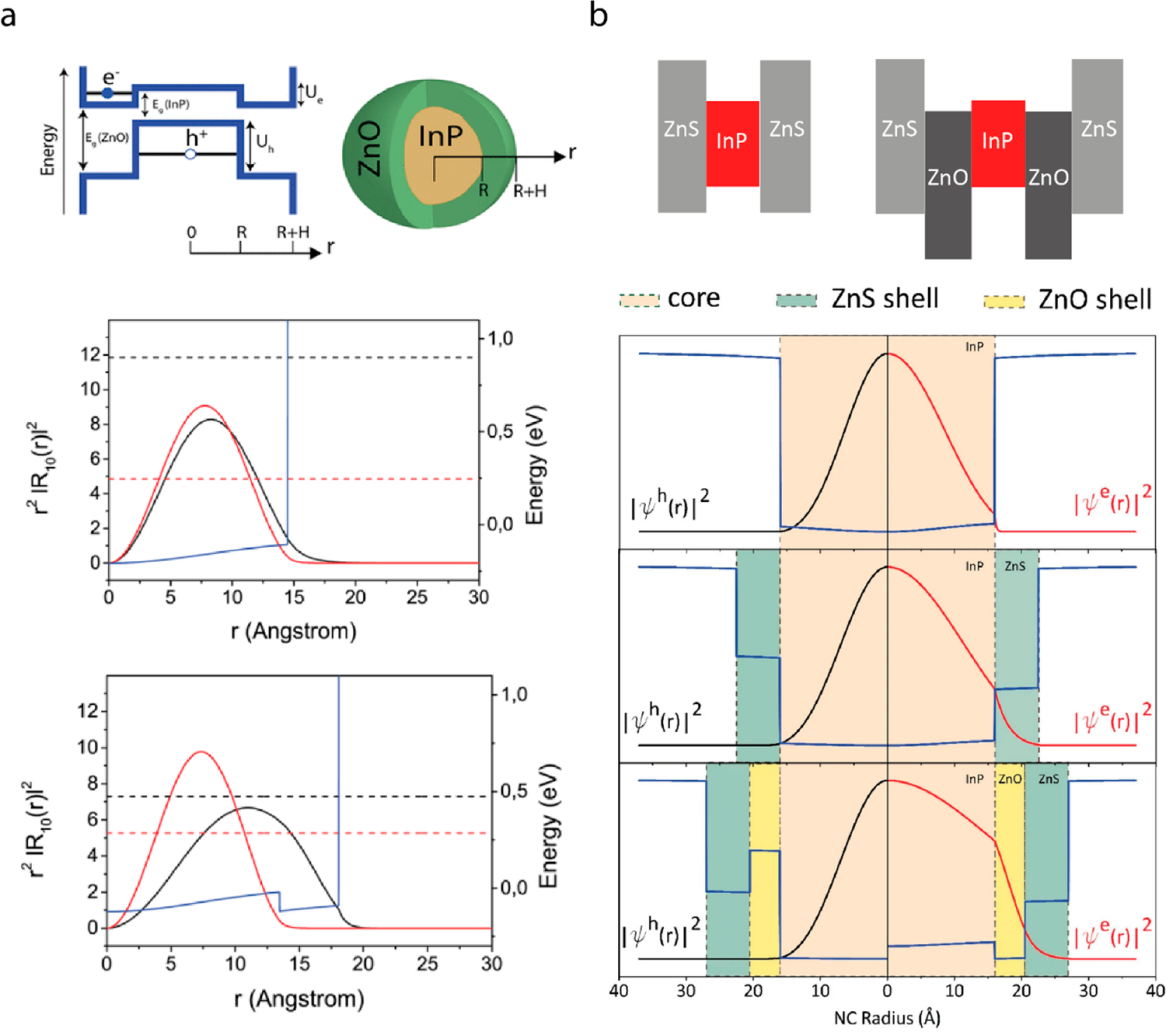
Quantum mechanical simulations of type I and type II nanocrystals, showing the effect of wave function engineering on charge carrier localization. (a) Top: Blue lines show energy band alignment and black lines show minimum electron and hole discrete energy levels. R and R+H correspond to the radius of the InP core and the InP/ZnO core/shell QDs, respectively. Bottom: Simulated electron and hole wave functions for the InP core (top) and the InP/ZnO core/shell (bottom) QDs. Black and red lines show electron and hole radial probability functions, respectively. Blue line represents the electron confinement potential. Dashed black and red lines represent single electron and hole energies, respectively. Reprinted with permission from ref (69). Copyright 2018 American Chemical Society. (b) Top: Energy band alignment schematics of type I InP/ZnS and type II InP/ZnO/ZnS QDs. Bottom: Simulated electron (red lines) and hole (black lines) wave functions for InP core, InP/ZnS core/shell, and InP/ZnO/ZnS core/shell/shell nanocrystals. Blue lines represent the electron confinement potential. Reprinted with permission from ref (83). Copyright 2021 Springer Nature. In both studies, type II nanostructures exhibit electron delocalization to shells, which leads to decreased electron–hole wave function overlap, i.e., reduced exciton binding energy.

Electron and hole localization within QD heterostructure influences the performance and/or charge injection mechanism of the QD-based biointerfaces. Remarkably, the carrier localization in QDs can be tuned by proper choice of core and shell materials. The conduction and valence band energy alignment of core and shell materials lead to formation of type I, quasi-type II, or type II QDs that can directly control the electron and hole wave functions, i.e., the spatial separation of electron–hole pairs (Figure 2c).^81^ Moreover, while keeping the heterojunction the same, the thickness of the shell also influences the carrier localization that shifts the oscillator strength of electronic transitions and thus the absorption profile.^82^ For neural interfaces, this nanoengineering ability at the single nanomaterial level is unique in comparison with their polymeric and metallic counterparts. For example, the photocurrent generation by a QD-based neural interface was shown to be enhanced when a type I QD (InP core) was replaced with its type II counterpart (InP/ZnO) in the same device architecture.^69^ One of the main factors leading this improvement was reduced electron–hole wave function overlap, which facilitates more effective charge transfer to the nearby electron acceptor layer due to increased spatial separation of charges in type II QD and reduced surface defects (Figure 4a). This ability (Figure 4b) provides a promising route for tuning the contribution of capacitive and faradaic charge injection processes at the device-electrolyte interface as well (Figure 10e, f).^83^

### 3.2 Biophysical Mechanisms for Modulating Neural Activity

**Figure 5:**
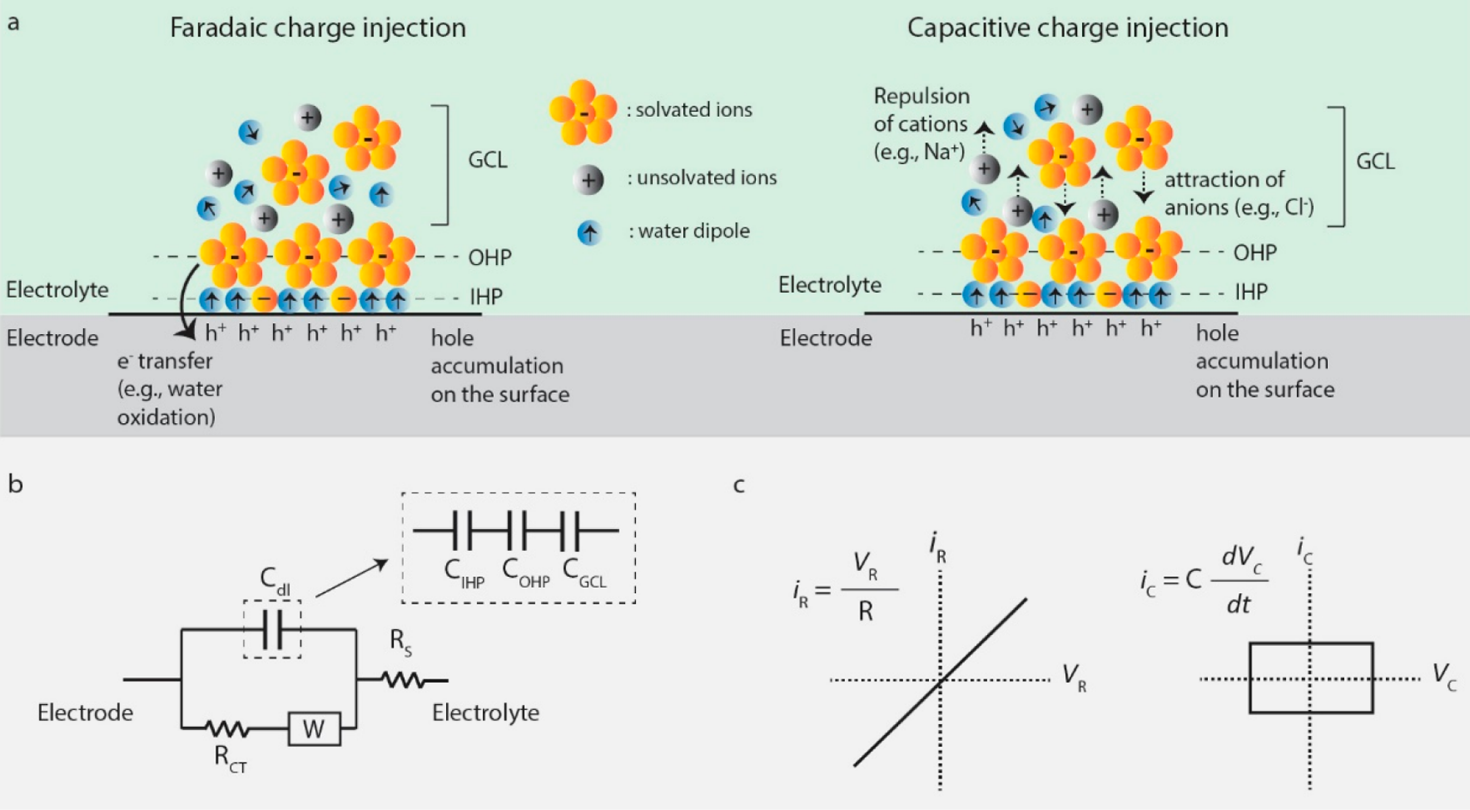
Primary charge injection mechanisms in QD-based biointerfaces. (a) Illustration of faradaic and capacitive charge injection mechanisms. Electrons or holes accumulate on the biointerface surface, inducing faradaic or capacitive charge injection in the electrolyte (hole accumulation was shown as a representative case). IHP, inner Helmholtz layer, OHP, outer Helmholtz layer, GCL, Gouy–Chapman diffuse-charge layer. (b) Electrical circuit model of the electrode–electrolyte interface. *C*_dl_ represents double-layer capacitance and is equivalent to the series sum of IHP, OHP, and GCL capacitances. *R*_CT_ and *R*_s_ denotes charge transfer resistance and solution resistance, respectively. *W* represents Warburg impedance. (c) Typical current–voltage profiles of resistive and capacitive elements.

In a QD-based neural interface, the photogenerated electrons or holes that accumulate close to the electrode/electrolyte interface induce electrochemical processes in the aqueous cellular environment. Those processes perturb the distribution of ions such as sodium, potassium, and chloride near the neural membrane, which leads to activation or suppression of neural activity. Depending on the interfacial impedance, one of the two primary charge injection mechanisms, faradaic or capacitive, take place at the electrode–electrolyte interface. These two mechanisms are illustrated in Figure 5a. In most cases, however, interfacial impedance involves both a double layer capacitance, a charge transfer resistance, and a Warburg impedance representing the diffusion processes in the presence of reversible reactions. Thus, a simple electrical circuit model consisting of a capacitor and a resistor can be used to model the electrode–electrolyte interface (Figure 5b).

#### 3.2.1 Faradaic Stimulation

The faradaic charge injection mechanism involves electron exchange between the neural interface and the electrolyte (Figure 5a). Charge transfer can take place in both ways, i.e., by injecting electrons into or extracting electrons from the electrolyte. Electron transfers at the electrode–electrolyte interfaces can lead to a wide variety of faradaic reactions. For example, electron injection into the electrolyte can cause reduction reactions (e.g., reactive oxygen species (ROS) generation), whereas electron removal from the electrolyte can lead to oxidation reactions. Thus, the direction of photocurrent gives information on the possible faradaic reactions occurring at the device–electrolyte interface. The occurrence of electron transfer between a neural interface and electrolyte depends mainly on two conditions. First, there should be a favorable energy state for electrons to move in the electrolyte. Second, the electric potential at the device/electrolyte interface should be in the range that is required for the occurrence of electron transfer reaction (e.g., see the review by Kumsa et al. for the detailed description of electron transfer processes between a stimulation electrode and electrolyte).^84^ When both conditions are satisfied and reactants are present at the electrode/electrolyte interface, reversible or irreversible faradaic reactions can arise.

In irreversible faradaic processes, the reaction product moves away from the reaction site faster than the electron transfer rate, meaning there is no charge storage at the interface and the reaction products cannot be reversed back to their reactant form.^85^ The unrecovered products diffusing into the electrolyte alter the physiochemical properties of the environment, posing potentially harmful effects to both neurons and biointerface. In this case, the dominant charge injection process is resistive, and the interfacial current–voltage (*IV*) profile in an ideal case, where double layer capacitor is negligible, can be represented with a resistor IV shown in Figure 5c.

Reversible faradaic reactions have much faster electron transfer rates compared to irreversible processes. Because the reaction products move away from the reaction site at a slower rate compared to the electron transfer rate, there is a charge storage in the interface in reversible faradaic reactions. Because of the closeness of reaction products to the interface, the products can be recovered back to their initial reactant form if the polarity of the electric potential is reversed.^85^ In an ideal case where the charge transfer resistance is infinite, this results in an *IV* profile of a capacitor at the electrode–electrolyte interface (Figure 5c).

Although the reduction oxidation processes occurring at the electrode–electrolyte interface have been studied for conventional electrodes like Au and Pt,^86,87^ redox processes at the QD–aCSF (or PBS) interface have not been elucidated yet. Dye-sensitized solar cells (DSSCs) are more mature technology with a similar operation principle to QD-based biointerfaces in the sense that they both operate in an electrochemical medium. However, the electrolytes used in DSSCs are different than aCSF or PBS. One recent study investigated the possible faradaic reactions occurring at InP QD-based biointerface-aCSF interface.^88^ The followed strategy was to investigate the photocurrent response of the biointerfaces in modified aCSF solutions, each is deficient of one constituent, to understand the faradaic processes according to the changes in the photocurrent in response to the removal of a constituent. This showed that the oxidation reactions at the QD–aCSF interface involve reactions with HEPES and water, while the reduction reactions are mostly occurring with water.^88^ Another possible strategy for identifying the faradaic processes could be the cyclic voltammetry (CV) analysis of QD biointerfaces in solutions of the constituent materials of aCSF or PBS.^87^ The resistive-like behaviors in *CV* measurements indicate the presence of faradaic reactions, and the corresponding potential values would enable identification of oxidized or reduced constituent.

#### 3.2.2 Capacitive Stimulation

When there is no net charge transfer between a neural interface and the electrolyte, photocurrent can be generated by redistribution of ions in the extracellular medium. Upon illumination of a QD-based biointerface, one type of charge carrier accumulates on the surface of the device, causing a change in the net charge of the surface. This induces the movement of oppositely charged ions close to the surface and similarly charged ions away from the surface, leading to formation of a double layer capacitor in the device/electrolyte interface. The double layer consists of three different layers, the inner Helmholtz plane (IHP), the outer Helmholtz plane (OHP), and the diffuse charge layer (Figure 5a). IHP is formed by adsorbed ions onto the surface and preferentially oriented water molecules forming a hydration sheath. The OHP mostly contains solvated ions that are not able to penetrate the hydration sheath. Finally, the diffuse layer contains both solvated and unsolvated ions whose density decreases with the distance from the electrode–electrolyte interface. Hence, the total double layer capacitance (*C*_dl_ in Figure 5b) is composed of serially connected IHP, OHP, and diffuse layer capacitances.^89^ Ideally, pure capacitive electrodes have infinite charge transfer resistance (*R*_CT_ in Figure 5b), meaning that the charge transfer rate is zero at the electrode–electrolyte interface and all current flows through the double layer capacitor as displacement current, which leads to a capacitor *IV* profile at the interface (Figure 5c).

Because capacitive stimulation involves a reversible charging/discharging process and does not cause electron exchange between the device and electrolyte, the physiochemical properties of the electrolyte such as electroneutrality and pH are preserved. This renders capacitive charge injection mechanism as a safer alternative to irreversible faradaic processes, which motivates researchers to introduce viable methods for tuning the capacitive and faradaic components of the injected charge to minimize the faradaic and maximize the capacitive charge injection.

#### 3.2.3 QD Biointerface Design for Controlling the Charge Injection Mechanism

**Figure 6:**
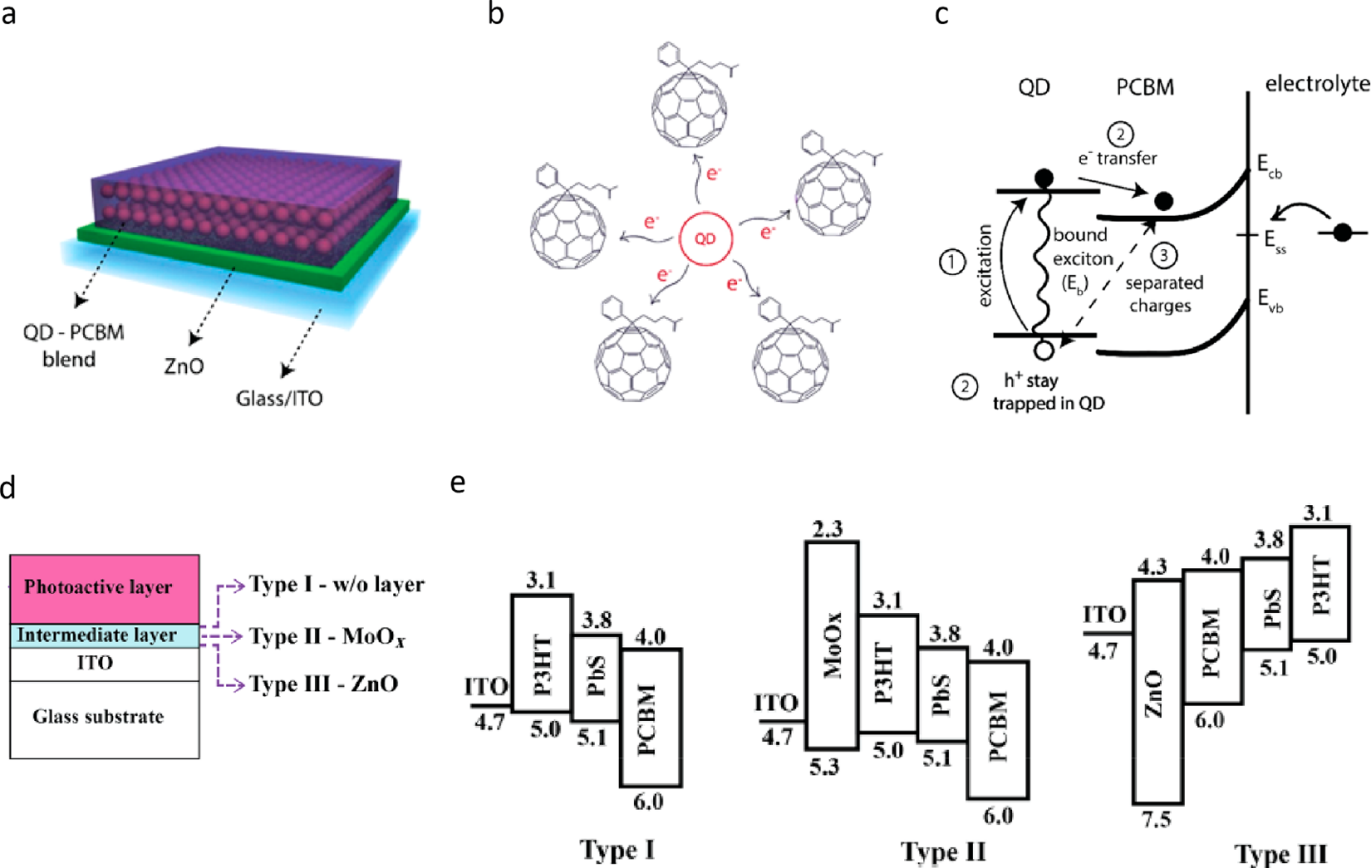
Obtaining capacitive-dominant QD-based biointerfaces via donor–acceptor nanoheterojunction (a–c) and band alignment engineering (d, e). (a) Device structure of QD-fullerene donor–acceptor nanoheterojunction-based interfaces for obtaining capacitive-dominant photoresponse. (b) Schematic showing the electron transfer from QD to fullerene derivative PCBM upon photoexcitation. (c) Capacitive photocurrent generation mechanism showing each step in a consecutive manner. *E*_b_, exciton binding energy; *E*_cb_, *E*_vb_, *E*_ss_, conduction band, valence band, and surface state energy levels, respectively. Band bending at the electrolyte interface prevents electron transfer to electrolyte. Electrons will then be transferred to ZnO and ITO because of the high electron mobility of ZnO. Holes are trapped in the QD valence band because of the ZnO valence band level, which induces capacitive photocurrent. Panels a, b, and c reprinted with permission from ref (83). Copyright 2021 Springer Nature. (d) Schematic of device architecture containing photoactive layers of P3HT:PbS:PCBM, with the intermediate layer (ZnO, MoOx, or none) coated on glass/ITO substrates. Although the photoactive layer generates excitons within the device, the energy level of the intermediate layer determines the surface polarity by routing either the electrons or the holes toward the top surface layer. (e) Manipulation of the band alignment via choice of different intermediate layers. Type I and type II devices generate faradaic-dominant photocurrents due to electron transfer from the PCBM LUMO level to electrolyte. Type III architecture accumulates holes on the surface. Holes do not interact with the electrolyte because of the unfavorable energy level of P3HT HOMO and water oxidation levels, which leads to capacitive-dominant photoresponse. Panels d and e reprinted with permission from ref (71). Copyright 2018 American Physical Society.

Because of the reversibility and biocompatibility of capacitive charge injection mechanism, different strategies were proposed to minimize the faradaic processes in QD-based neural interfaces to generate a capacitive-dominant photoresponse. Inspired from donor–acceptor bulk heterojunctions used in organic solar cells, QDs were used as a donor material in a QD–fullerene nanoheterojunction structure to produce capacitive-dominant photocurrents in an extracellular medium.^83^ QD–fullerene donor–acceptor structure separates the photogenerated electron–hole pairs, whereas the ZnO layer in the device architecture facilitates further separation by providing high mobility to electrons (Figure 6a, b). In addition, to minimize the electron transfer between the surface traps and electrolyte (Figure 6c), the PLQY of the QDs was kept high, which is an indication of successful surface passivation.^85^ Another study demonstrated the control of faradaic and capacitive processes to the photoresponse of a biointerface by engineering the electronic band alignment of the device without surface modification.^71^ Proper manipulation of the band alignment enables controlling the type of charge carrier (electron or hole) that will be accumulated on the electrolyte interface (Figure 6d, e). If the energy level of the charge carrier accumulated on the surface is not favorable for involving in faradaic reactions, then the electron transfer between the biointerface and electrolyte is minimized. The accumulated charge carrier then induces capacitive photocurrent by charging/discharging of the double layer formed at the interface.^71^

So far, all the reported QD-based biointerfaces are optoelectronic devices, i.e., they transduce electromagnetic energy into electrochemical current through photocapacitive or photofaradaic mechanisms, thereby electrically stimulating neurons. Light can also be converted into thermal or acoustic energy via photothermal and photoacoustic effects to modulate the neural activity. Absorption of light can trigger lattice vibrations in a crystal structure as a result of nonradiative phonon processes. These vibrations generate local transient heat, which can evoke action potentials by opening thermosensitive ion channels^90^ or causing capacitive membrane currents.^91^ Alternatively, the induced transient heat upon photoexcitation can cause acoustic wave generation by thermoelastic expansion and contraction of molecules in the photoexcited region, which can mechanically stimulate neurons.^92^ Different material types such as silicon,^93^ metallic nanoparticles,^94^ graphene,^95^ and organic polymers^96^ showed promise for utilizing photothermal effect for building effective neurostimulation systems. The use of photoacoustic effect for neurostimulation was more recently introduced and reported in only a few studies so far.^97,98^ Such alternative stimulation mechanisms can be exploited by next-generation QD-based biointerfaces to design alternative systems involving QDs in transducing optical energy into thermal and acoustic energies.^99,100^

To date, optoelectronic QD–biointerfaces have used different II–VI and III–V semiconductors such as mercury telluride (HgTe), cadmium selenide (CdSe), indium phosphide (InP), lead sulfide (PbS), and aluminum antimonide (AlSb), which are summarized in Table 1. In the next section, we examine all these structures in detail, compare their disadvantages and advantages, and draw a perspective for future studies.

**Table 1:** Examples of Semiconductor Nanoparticle-Based Optoelectronic Neural Interfaces^a^

nanoparticle	interface type	dominant charge generation	modulation effect	operational illumination intensity (mW cm^–2^)	responsivity (mA/W)	cell type	transmembrane potential change (mV)	refs
HgTe	multilayered	capacitive	excitatory	800	N/A	NG108	+10	(67)
	ITO/(PDDA/HgTe)_N_	faradaic						
CdSe	single-layer	N/A	excitatory	0.46	N/A	LnCap	+13	(68)
CdTe	CdSe and CdTe QD films	N/A	inhibitory				–4	
CdSe/CdS	multilayered							(113)
	CNT CdSe/CdS	capacitive	excitatory	30	0.6	embryonic chick retinas, E14	N/A	
InP/ZnO	multilayered		excitatory	0.40	N/A	PC12	+45	(69)
	ITO/ZnO/InP//ZnO QD	faradaic						
InP/ZnS	multilayered		excitatory	57	2.3	PHN	+110	(88)
	ITO/InP//ZnS QD/ZnO	faradaic	inhibitory		7.5		–45.6	
	ITO/TiO_2_/InP//ZnS QD							
InP/ZnO/ZnS	multilayered		N/A	57	0.8	N/A	N/A	(83)
	ITO/ZnO/PBCM:InP/ZnO QD	capacitive						
InP/ZnS QF	multilayered		excitatory	169	N/A	SH-SY5Y	+1.21	(70)
	ITO/TiO_2_/InP//ZnO QDs	faradaic						
PbS	multilayered		excitatory	1	N/A	SH-SY5Y	+4.7	(71)
	ITO/P3HT:PbS QD:PCBM	faradaic						
	ITO/MoO_*x*_/P3HT:PbS QD:PCBM	faradaic						
	ITO/ZnO/P3HT:PbS QD:PCBM	capacitive						
PbS	multilayered		excitatory	1	99	PHN	+70	(101)
	ITO/ZnO/P3HT:PbS QD:PCBM	capacitive						
AlSb	multilayered		excitatory	100	6	PHN	+103	(102)
	ITO/ZnO/P3HT/AlSb QD	capacitive						

a / indicates layer-by-layer coating, whereas // indicates core/shell QD structures.

## 4 Quantum Dot Systems for Neural Stimulation

### 4.1 HgTe QD-Based Neural Interfaces

**Figure 7:**
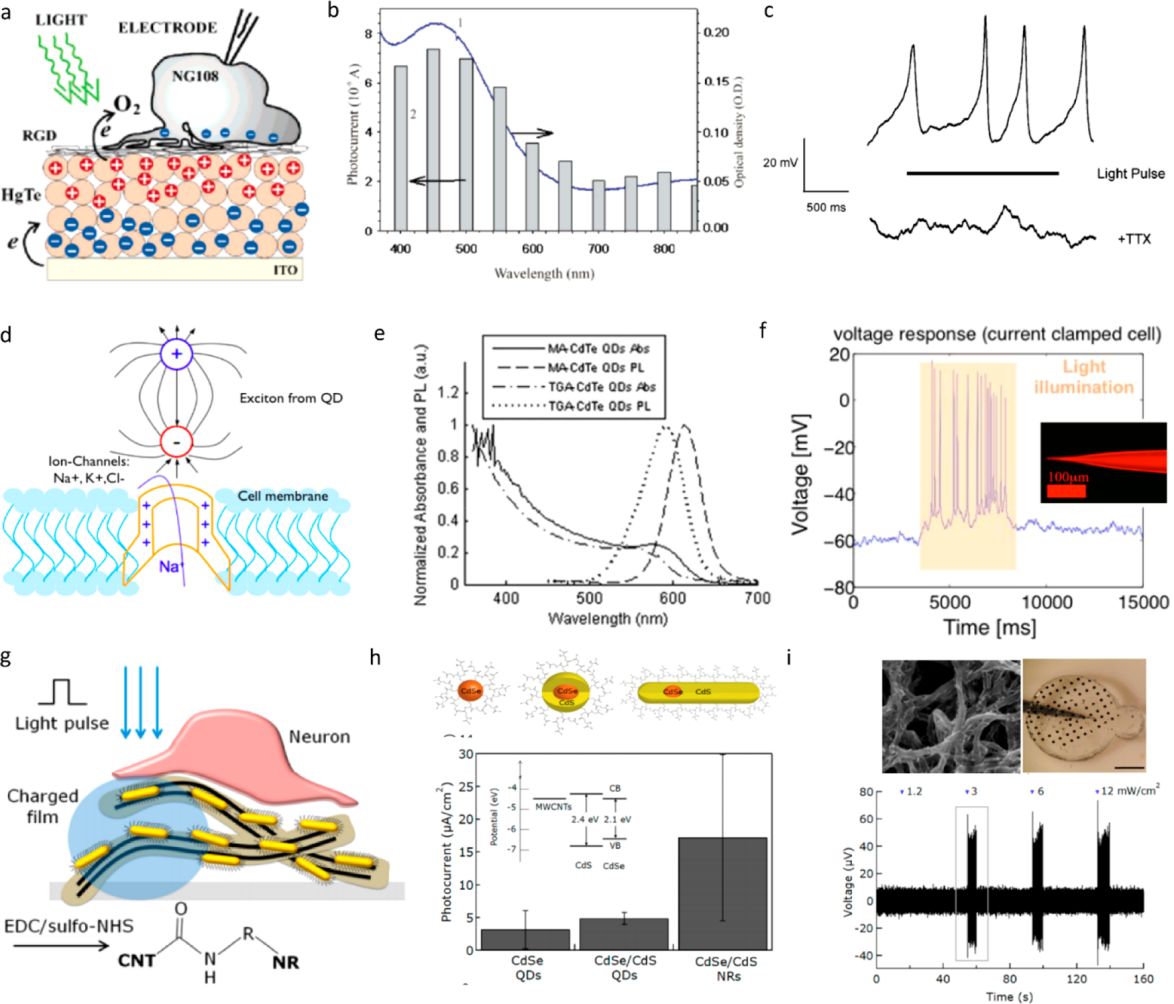
Pioneering semiconductor nanoparticle-based optoelectronic neural interfaces. (a) HgTe QDs stabilized with thioglycolic acid-coated single-material device. (b) Light absorption characteristics (1, solid line) and photogenerated voltage (2, bars) of HgTe QDs and layer-by-layer films. UV–vis absorption spectrum on HgTe QD dispersion stabilized by thioglycerol used for fabrication of LBL films. (c) Action potential responses of NG108 cells grown on (PDDA/HgTe)_12_ + (PDDA/Clay)_2_ under photostimulus with and without tetrodotoxin (TTX). Panels a, b, and c reprinted with permission from ref (67). Copyright 2007 American Chemcial Society. (d) Schematic of the interaction between a QD and cell membrane. (e) UV–visible absorbance and photoluminescence (PL) characterization of CdTe QDs. (f) Current-clamped recording of cortical neurons on CdSe QD film. Fluorescence image of a micropipette coated with CdSe QDs used for single-cell stimulation. Panels d, e, and f reprinted with permission from ref (68). Copyright 2012 The Optical Society. (g) Schematic of the optoelectronic coupling between NR-conjugated CNT coated by ppAA. (h) Schematic drawing of the CdSe–GSH QDs (left), CdSe/CdS–GSH QDs (center), and CdSe/CdS–GSH NRs (right). Average photocurrents for different devices based on CdSe, CdSe/CdS, and CdSe/CdS NRs with CNTs under an excitation pulse of 30 mW cm^–2^ for 100 ms with a 405 nm illumination source. (i) (Upper left) SEM image of an NR–CNT film (scale bar: 100 nm). (Upper right) CNT electrode array on a PDMS flexible support (scale bar: 1 mm). (Bottom) Extracellular voltage trace recorded from a chick retina following 100 ms light stimulation (405 nm, pulse interval of 30 ms) under different intensities (1.2, 3, 6, and 12 mW cm^–2^). Panels g, h, and I reprinted with permission from ref (113). Copyright 2014 American Chemical Society

During the rise of colloidal quantum dots in the 1990s, superior properties such as high sensitivity and responsivity of QDs have been utilized by scalable production techniques in several optoelectronic devices. Among them, HgTe QDs took particular attention as a narrow semimetal absorber and they were widely studied for photodetectors.^103−105^ The pioneering work of Kotov and his co-workers^67^ demonstrated the first photostimulation of neurons by using QDs, for which HgTe QD was used. The whole device structure with HgTe and PDDA layers was fabricated via layer-by-layer (LBL) on conductive ITO-coated glass substrates, where the ITO layer supplies electrons to the system (Figure 7a). HgTe QDs were stabilized with thioglycolic acid for surface passivation and coated with poly(dimethyldiallylammonium chloride) (PDDA) as a positively charged partner of the HgTe QDs (Figure 7a).^67^

Upon excitation with visible illumination (Figure 7b), the photogenerated excitons in the HgTe layer show a single photon absorption process since the photocurrent increases linearly with the increasing illumination intensity. The origin of photocurrent is attributed to the photoinduced electron transfer between the HgTe QD layer and O_2_ because the HgTe QD conduction band is at −4.6 eV and O_2_ acceptor energy level is at −5.3 eV. Moreover, the biphasic behavior of the photocurrent transient indicates a capacitive pathway due to separate charging and discharging peaks. To evaluate the biological response, we chose neuroblastoma-glioma cell line NG108 as the model cell line, which benefits from being more resistant to environmental changes. As a solution for providing a biocompatible and adhesive layer for cell attachment against the toxic-heavy-metal Hg content, polylysine/poly(acrylic acid)/polylysine (PLP) was adopted as the interfacial layer. The membrane potential of cells were recorded with a patch-clamp system, which enables the measurement at the single-cell level, and successful stimulation of cells was observed (Figure 7c). In some of the coupled individual cells, 10 mV depolarization was observed while the mean depolarization was 2.3 ± 2.4 mV due to coupling issues between the cells and the biointerface. The membrane depolarization levels after several stimulations showed minimal membrane resistance change, indicating safe stimulation without thermal or direct light gating effects. To support these biocompatibility signs, we also added LBL films of clay sheets as the interfacial layer. Although clay enabled capacitive currents, during the electrophysiology experiments, faradaic (resistive) coupling with cells was interestingly observed. This pioneering study opened a new direction of research by using QDs for modulation of the electrical activity of cells, and the results also point out the requirement of biocompatible and capacitive neural interfaces for cell stimulation.

### 4.2 Cd-Based QD Neural Interfaces

After the initial study on the HgTe QD-based biointerface, the work by Lugo et al.^68^ suggested a new theoretical framework based on the near-field electromagnetic wave and membrane coupling. They proposed the electric dipole moment created by the electron–hole separation in the excited QD with the membrane potential change (Figure 7d). The relation between the QD-induced electric field and membrane proximity can be regarded as a superposition of each electron–hole pair generated by the QDs.^68^ Considering the Debye length of the saline, the electric field potential may drop exponentially with the distance between the QDs and the cell membrane. Motivated by combining this theoretical framework with experimental results, they have used multilayer QD films, specifically CdTe and CdSe films, without any other mediator layer. Cd-based QDs were already widely used in fluorescent imaging studies in vivo^106,107^ and biological labeling in vitro^108−111^ because of their strong absorption and emission in the visible spectrum (Figure 7e). Electrophysiology experiments on cultured prostate cancer (LnCap) cells on CdTe QD films and cultured cortical neurons on CdSe QD films showed promising results. Under 430 nm with 1 × 10^7^ photons μm^–2^ s^–1^ illumination, an equivalent of 462 μW cm^–2^, CdTe QD films induced membrane hyperpolarization, which is attributed to the activation of potassium channels in prostate cancer cells.^112^ Moreover, the cells that are 20–30 μm above the CdTe QD film were not affected by the electric field because of the exponential decay of the field with distance. The optoelectronic characterization, particularly photocurrent measurements without the seeded cells, would be useful to distinguish any contribution by the Faradaic reactions. Second, CdSe QD films were tested with cortical neurons under 550 nm illumination with the same intensity. The excitation of the QD films led to membrane depolarization and evoked multiple action potentials (Figure 7f). However, in both studies, there were temporal and spatial variations in the stimulation performance. This interesting study suggested an alternative mechanism to modulate membrane potential via QDs.

To improve the optoelectronic performance of biointerfaces, Hanein and her co-workers combined two nanomaterial systems, namely, semiconductor nanorods (NRs) and carbon nanotubes (CNTs) (Figure 7g).^113^ The latter is already proven for its neural recording and stimulation capabilities^114,115^ because of their high surface roughness, highly porous nature, and large capacitance at the interface, and the former is the convenient choice for efficient and tunable light absorption. Moreover, the combination of two such systems led to improved charge separation and enhanced optoelectronic performance in comparison with previous studies. To motivate the use of NRs, wecompared three different nanocrystals (NCs), namely, CdSe QDs, CdSe/CdS core/shell QDs, and CdSe/CdS NRs (Figure 7h). As most of the colloidal QD synthesis ends up with nonpolar solvents such as hexane, toluene, and chloroform, it is critically important and a major challenge to render these NCs in an aqueous solution. Because of the advantage of ligand engineering of these NCs, the original ligands were replaced with the antioxidant tripeptide–glutathione (GSH),^113^ which is also beneficial for the aqueous stability and biocompatibility, as it reduces the release of cadmium ions to the solution. CdSe, CdSe/CdS QDs, and CdSe/CdS NRs were all coated with GSH and conjugated with CNT films. CdSe GSH and CdSe/CdS GSH systems required higher loading concentrations than the CdSe/CdS NRs, approximately 9 × 10^13^, 1.75 × 10^13^, and 0.75 × 10^13^ particles cm^–2^, respectively.^113^ In addition to the lower concentration, CdSe/CdS NRs generated much higher photocurrents (Figure 7h) because of their large surface area for efficient charge separation and high coupling with CNTs that was due to proper energy level alignment. Moreover, to evaluate the neural stimulation performance, they utilized light-insensitive embryonic chick retinas (E14). Retina or primary neurons are suitable application targets for neural interfaces working in the visible window because the eye itself is highly transparent in these wavelengths, suitable for wireless excitation mechanisms. The particular choice of the E14 stage is important because retinal cells are at the early maturation stage, and photoreceptors are not developed.^113,116^ Excitation with 100 ms, 405 nm pulses revealed electrical response, and the intensity threshold of 3 mW cm^–2^ (Figure 7i) indicates the potential use of the biointerface under ambient light intensities.^113^ The illumination intensity of the excitation and the exposure duration are particularly important for applications targeting the eye and brain. Light exposure above threshold intensities and exposure times may lead to thermal, thermoacoustic, and photochemical damage both in the targeted area as well as in the nearby tissues.^117,118^ In comparison with previous studies, the combination of NR-CNT nanomaterial systems significantly reduces the threshold light intensity (Table 1) because effective charge generation and separation can be achieved via improved conjugation and proper band alignment. Therefore, this novel approach inspired various studies combining not only NRs with CNTs but also different semiconductor NCs with organic polymers and 2D/3D materials.

### 4.3 InP QD-Based Neural Interfaces

Previous efforts for designing effective neural interfaces had concentrated on cadmium^68^ and mercury-based^67^ QDs, following the chronological evolution of colloidal quantum dots. Alternatively, InP-based QDs are the improved candidates for neural interfaces in terms of lower cytotoxicity while having a high degree of tunable optical properties due to a large Bohr exciton radius (∼9 nm).^119^ Moreover, European Union research on exposure of nanomaterials, NANOMICEX, provides the guidelines for future development of less-toxic, environmentally friendly nanoparticles for commercial and biomedical applications, for which InP-based ones hold a great promise. Although InP QDs in either core or core/shell structures were extensively studied in several different fields including solar cells,^120−123^ fluorescent imaging markers,^120−123^ bioconjugated sensors,^124^ detectors,^125^ luminescent solar concentrators,^126−129^ and LEDs,^10,130,131^ their potential in neural interfaces remained unrevealed. On the other hand, their biocompatibility for both in vitro and in vivo studies was carefully studied in the literature,^132^ which they were used as optical probes for imaging and as nanocarriers for drug delivery applications.^133^

**Figure 8:**
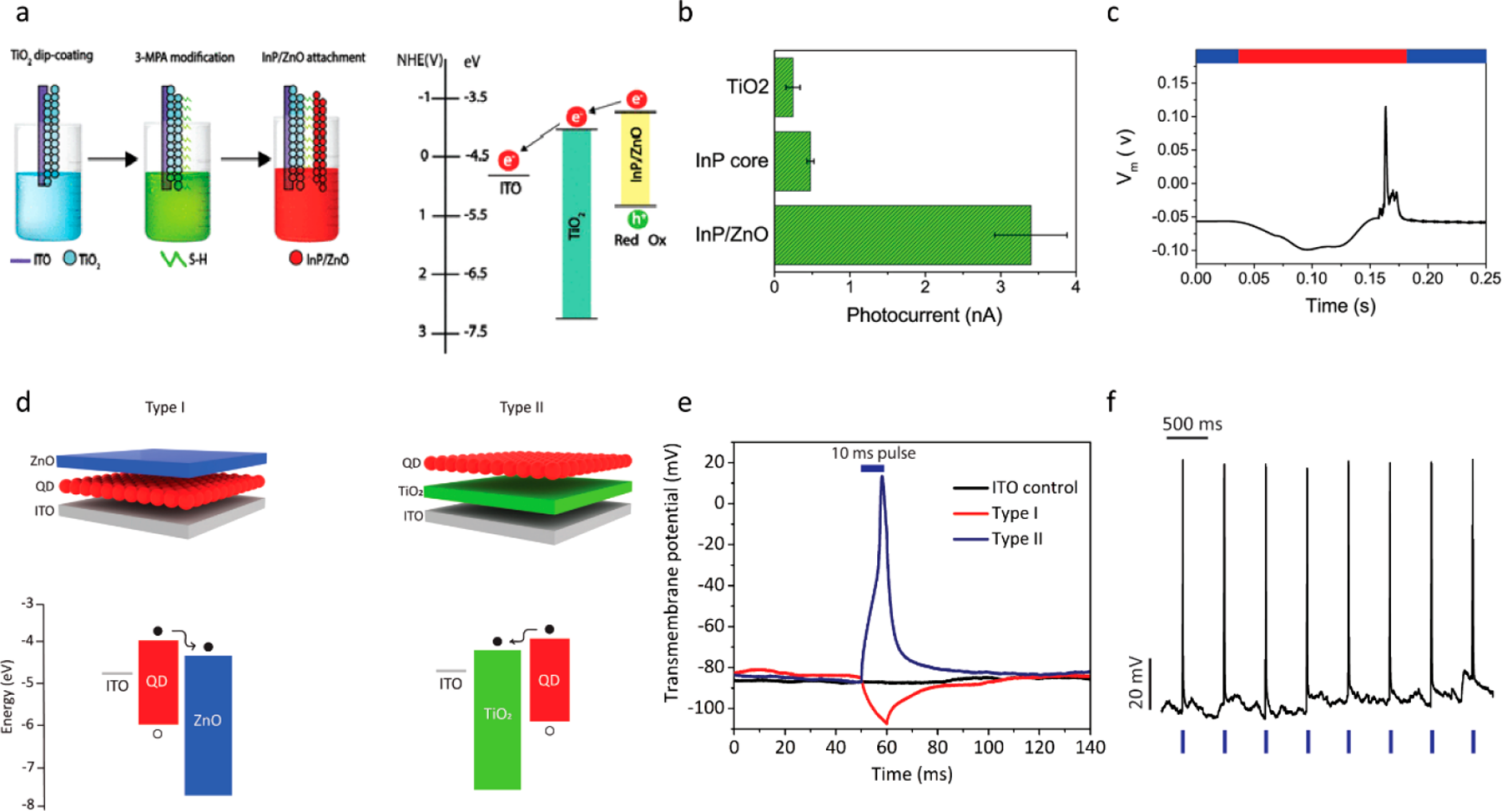
InP QD-based optoelectronic neural interfaces. (a) Schematic illustration of the photoelectrode fabrication steps and energy band diagram of the device architecture. (b) Photocurrent performance of TiO_2_, InP core and InP/ZnO QD coated biointerfaces. (c) Photostimulation of a PC12 cell on the photoelectrode under 4 μW mm^–2^ illumination (red bar, time period under illumination; blue bar, no illumination). Panels a, b and c reprinted with permission from ref (69). Copyright 2018 American Chemical Society. (d) Energy band diagram of bidirectional device architectures. (e) TEM image of the InP/ZnS QDs. (f) Transmembrane potential recordings of neurons on type I, type II, and ITO control samples (illumination: blue LED at 445 nm, 10 ms pulse width, 2 mW mm^–2^ optical power density; blue bar indicates the 10 ms “light on” interval). Panels d, e, and f reprinted with permission from ref (88). Copyright 2021 Frontiers.

The InP core material is a standard nanostructure that can be used for light-to-charge conversion, whereas core/shell heterostructures with type II band alignment have attractive properties for optoelectronic applications owing to the ability to control the spatial confinement regimes of charge carriers throughout the core and shell materials. In this context, core/shell structures with less toxic materials have been favorably used for photostimulation applications. The first study incorporating type II QDs, namely, InP/ZnO core/shell QDs (in thin-film layered configuration), showed its high potential for neural interfaces by generating hyperpolarization of the cell membrane.^69^ The crystalline ZnO shell builds the type II structure because of its wide bandgap (3.37 eV)^134^ and similar conduction band level with InP, while protecting the core material from oxidative reactions, just like ZnS shelling of CdSe core QDs discussed above. For the shell growth, thermal decomposition of zinc acetylacetonate was utilized by heating the solution in reaction.^128^ To promote the optoelectronic performance, we integrated InP/ZnO QDs onto a photoelectrode structure of glass:ITO/TiO_2_ to generate extracellular currents for photostimulation (Figure 8a left). The particular choice of TiO_2_ nanoparticles modified with 3-mercaptopropionic acid (3-MPA) facilitates the binding of InP/ZnO QDs on TiO_2_ thin film. Upon illumination, photogenerated electron–hole pairs dissociate to core and shell materials. The strong interparticle interaction between InP/ZnO-TiO_2_ materials is due to the proximity between these layers by the short-chain linker molecule^135^ and this strategy couples the electron to an available state in TiO_2_. The coupled excited electron then diffuses toward the ITO layer, generating the photocurrent (Figure 8a right). The charge transfer between InP/ZnO QDs and TiO_2_ NPs led to a decreased average recombination lifetime of InP/ZnO QDs.^69^ The optoelectronic measurements showed ∼3.4 nA photocurrent generation (Figure 8b) under a low light intensity of 4 μW cm^–2^ at 445 nm, which is 26-fold lower than the ocular safety limit^117^ under pulsed illumination. Moreover, biocompatibility measurements with MTT for mitochondrial activity and LDH assay for membrane integrity of Neuro2A cells suggest high biocompatibility. The patch-clamp electrophysiology experiments on PC12 cells grown on the biointerface showed membrane hyperpolarization of −45 ± 10 mV under the same illumination intensity and evoked hyperpolarization-induced action potential, also called anode break excitation (Figure 8c), which is higher than the previous Cd^67^ and He^68^ QD-based studies at the same light intensity levels.

To date, most of the QD-based biointerface designs for neural stimulation concentrated on the depolarization of the cell membrane as a merit of success, which is the first step for generating neural activation. In contrast, silencing the neural activity by hyperpolarization of neurons was also proven to be effective for certain neurological disorders such as epilepsy.^136^ Moreover, achieving reliable and reversible inhibition of neurons enables systemic analysis of the cellular networks, which is highly motivated by neuroscientists.^137^ Therefore, the development of neural interfaces that can control depolarization and hyperpolarization can bring a new perspective and add versatility to neural therapeutics. In this context, a recent study by Karatum et al.^88^ utilized InP/ZnS core/shell QDs and metal oxide nanoparticles to design two different photovoltaic architectures, called type I (ITO/InP//ZnS QD/ZnO) and type II (ITO/TiO_2_/InP//ZnS QD) that can hyperpolarize and depolarize the neurons and lead to bidirectional control of the neural activity. Similar to recent studies,^34,132,138^ ZnO and TiO_2_ NPs were chosen as hole blockers because their HOMO levels. The band alignment in these designs drifts photogenerated electrons toward the interfacial layer in type I device generating anodic photocurrents and toward the ITO layer in type II device generating cathodic photocurrents (Figure 8d). Therefore, InP/ZnS QDs were used for injecting anodic and cathodic currents to the biological medium, inducing either hyperpolarization or depolarization of the neural membrane, respectively (Figure 8e). Favorably, both types can elicit more than 25 mV photovoltage under low light intensities (10 mW cm^–2^).^88^ For intensities as high as 57 mW cm^–2^, which is still lower than the threshold for thermal effects, type I and type II devices can produce −65 ± 7 mV and 175 ± 13 mV, respectively. Moreover, total charge injection in one charging/discharging phase was calculated as 1.29 μC cm^–2^ for type I and 4.12 μC cm^–2^ for type II biointerfaces. The generated photovoltage and charge injection levels are at similar levels with the required thresholds for neural stimulation.^40^ Different from previous studies, photoactive layer thickness, which is a crucial aspect for designing photovoltaics and extensively studied by solar cell research,^139−141^ was investigated in terms of depletion width and minority carrier diffusion length.^88^ The sum of diffusion length and depletion width, which provides the required thickness for increasing charge extraction efficiency and harvesting of these charge carriers, is ∼165 nm and ∼185 nm for type I and type II devices, respectively. This analysis is particularly useful to determine the required layer thickness for the photoactive material and guide the researchers to design more efficient devices. Moreover, primary hippocampal neurons (PHNs) grown on the optimized devices were tested with MTT assay, indicating high cell viability. The electrophysiology experiments showed ∼50 mV hyperpolarization for type I biointerfaces and successful neural activation for different frequency stimuli (1, 2, 5, and 10 Hz) for type II devices with faradaic mechanisms under 2 mW mm^–2^, 445 nm illumination (Figure 8f). Therefore, this study showed the ability to control the direction of stimulation with systematic engineering of band alignment and nanostructures with potential device performance to activate or suppress the neural activity of primary cells.^142^

**Figure 9:**
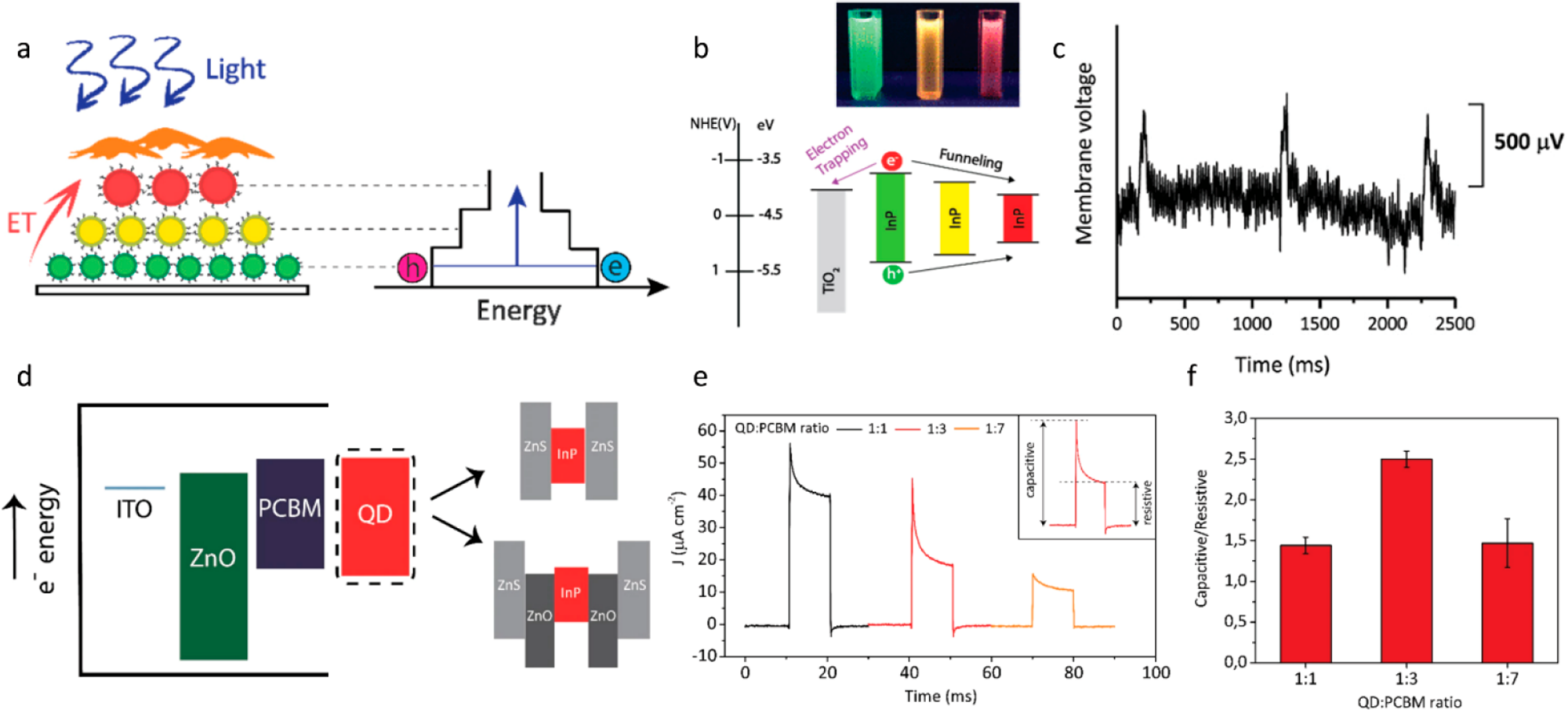
(a) Artificial antenna complexes made of rainbow InP quantum dots showing nonradiative energy transfer toward the cell interface. (b) (Upper inset) Photograph of the colloidal green-, yellow-, and red-emitting QDs under UV illumination. (Bottom) Energy band diagram of the quantum funnel biointerface (c) Photostimulation of the SH-SY5Y cell on the quantum funnel biointerface under illumination of 169 mW cm^–2^ with 50 ms illumination pulses. Panels a, b, and c reprinted with permission from ref (70). Copyright 2019 American Chemical Society. (d) Energy band alignment of the QD integrated biointerface. InP/ZnS core/shell and InP/ZnO/ZnS core/shell/shell QDs were incorporated into the photoelectrode architecture. (e) Photocurrent density traces of the devices with InP/ZnO/ZnS:PCBM volume ratios of 1:1 (black), 1:3 (red), and 1:7 (orange). The inset shows the components of the photocurrent. Capacitive current is the peak photocurrent reached after the light onset, whereas resistive current is the photocurrent remained after 90% of the illumination duration has passed. (f) Ratios of the capacitive to resistive components for devices with different QD:PCBM mixing ratios. Panels d, e, and f reprinted with permission from ref (83). Copyright 2021 Springer Nature.

In addition to the regular photovoltaic device architecture, inspired from the dipole–dipole interaction so-called Förster energy transfer in biological systems, graded quantum dot systems, (i.e., also called as rainbow quantum dots),^143^ can be utilized for building artificial antenna complexes just like photosynthetic systems (Figure 9a).^144^ As motivated by the reduced toxicity of InP QDs, biocompatible quantum funnels were studied for directing light-induced charges to the device/cell interface. The study by Bahmani Jalali et al.^70^ showed the synthesis and fabrication of multilayers of green-, yellow-, and red-emitting QDs in a graded structure to enable near-field dipole–dipole interaction (Figure 9b). QDs were engineered to achieve spectral overlap between the emission of the smaller QDs with the absorption spectrum of the larger QDs for efficient energy transfer toward the largest QD in the system. In the end of this excitonic transfer, the exciton dissociation is achieved via hole capturing by the S^2–^ groups of the 3-MPA ligand and induced faradaic currents for membrane potential modulation. InP cores were coated with sufficiently thin ZnS shells for all green-, yellow-, and red-emitting QDs by a hot injection method that allow efficient dipole–dipole coupling between the energy gradient QDs.^145,146^ The ZnS shell is important for both surface passivation of the defect states and to increase the quantum yield since higher QY offers an increase in Förster radius,^147^ which enhances the nonradiative excitonic energy transfer efficiency. The passivation of the defect states also plays a significant role in photocurrent generation because it reduces the midgap state trapping of excited electrons. A careful fabrication strategy is required for this type of graded funnel structure because one donor QD can transfer its excited energy to three acceptor QDs located nearby the donor.^147^ To facilitate this, we assembled three layers of red-emitting QDs on top of the green and yellow QD films with thicknesses of 14–24 nm^70^ (Figure 9a).^148^ The quantum funnel effect is proven by the optical characterizations showing weak photoluminescence in the green-yellow region followed by strong emission in the red spectral window, suggesting energy transfer from the donor QDs to red InP/ZnS acceptor QDs. Although the quantum funnel device showed lower absorption, its emission is stronger than the ungraded device, which was attributed to the trapped exciton recycling by energy transfer.^149^ Likewise, this graded quantum funnel structure generated a higher photocurrent than the ungraded control structure under 450 nm 169 mW cm^–2^, 500 ms pulsed illumination. The graded quantum funnel device also performed better under low light intensity and shorter illumination pulses. Advantageously, MTT and LHD assays on SH-SY5Y cells indicated minimal effects on cell viability and membrane permeability. Therefore, the designed biointerface showed a high potential for neural stimulation. The single-cell patch-clamp electrophysiology experiments indicated the induced up to 1.5 mV membrane depolarization via 1s pulses and even generated ∼500 μV depolarization under the same light intensity with shorter 50 ms pulses (Figure 9c). The performance loss with shorter pulses is generally expected because the charging time required for depolarization is also shorter. One disadvantage of the system is the dependence on faradaic charge generation, which needs to be carefully controlled for potentially harmful effects on the cellular environment, and charge injection performance should be significantly increased. Nevertheless, this unconventional design using nanophotonics may bring a new perspective to the field for novel biointerface designs.

**Figure 10:**
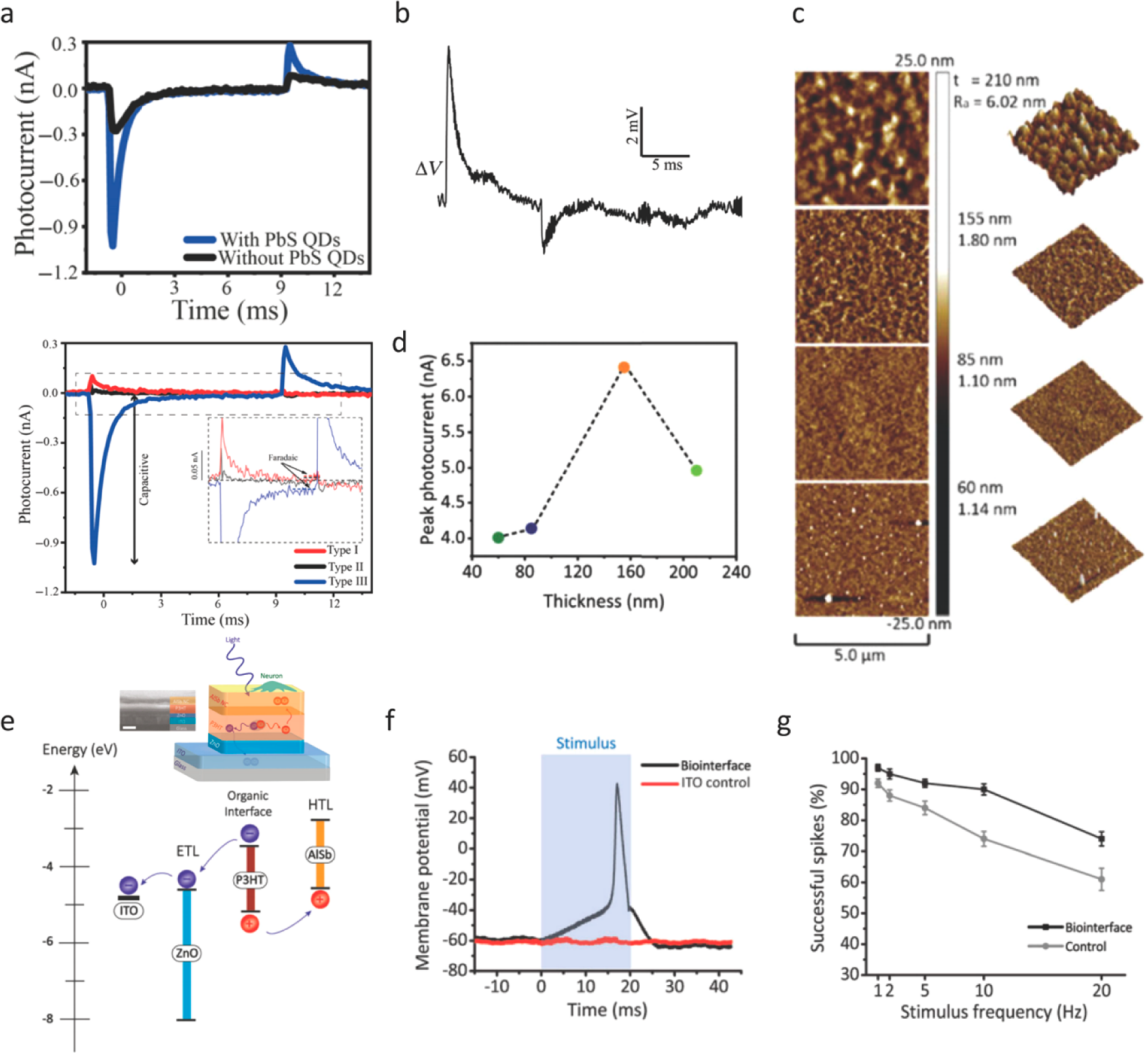
PbS- and AlSb-based neural interfaces. (a) (Top) Photocapacitive current levels of ITO/ZnO/P3HT:PCBM and ITO/ZnO/P3HT:PbS-QDs:PCBM photoelectrodes. (Bottom) Capacitive and faradaic components of type I, type II, and type III photoelectrodes under illumination of 10 ms light pulses with an intensity of 1 mW cm^–2^. The architecture for different types of biointerfaces was explained in panels c and d in Figure 6. (b) Membrane potential variation of SH-SY5Y cells grown on the type III biointerface in panel d upon light illumination (10 ms, 1 mW cm^–2^). Panels a and b reprinted with permission from ref (71). Copyright 2019 American Physical Society. (c) Atomic force microscopy (5 μm × 5 μm) of P3HT:PCBM surfaces with the optimized binary ratio of 2:1 on ITO/ZnO-coated glass substrates (left, 2D views; right, 3D views) with various thin film thicknesses (*t*) in tapping-mode. *R*_a_ shows the average surface roughness. (d) Peak photocurrent for the binary photoelectrodes as a function of various thin film thicknesses. Panels c and d reprinted with permission from ref (101). Copyright 2020 The Optical Society. (e) Structure of the AlSb integrated biointerface (left inset: cross-sectional SEM image) and energy band diagram of the proposed device. (f) Intracellular membrane potential change with respect to a distant Ag/AgCl electrode was measured after the photostimulation of primary hippocampal neurons on the glass:ITO control (red) and the biointerface (black) under illumination of 100 mW cm^–2^ with 20 ms illumination pulses. Blue semitransparent area shows the 445 nm light illumination period (g) Successful spike ratio of neurons on the glass:ITO/ZnO/P3HT control (gray) and the biointerface (black) under different illumination frequencies of 20 ms, 50 mW cm^–2^, and 20 pulses (*n* = 20, mean ± s.d.). Panels e, f and g reprinted with permission from ref (102). Copyright 2021 Springer Nature.

In the aforementioned studies, the dominant physical phenomena for photocurrent generation was generally faradaic by nature. However, irreversible faradaic charge injection is not desired for long-term and safe cellular stimulation. To suppress the photoelectrochemical charge transfer and increase device efficiency, researchers proposed a QD–fullerene donor–acceptor nanoheterojunction.^83^ In the study, a capacitive-dominant photoresponse was achieved using InP/ZnO/ZnS QDs and a fullerene derivative of [6,6]-phenyl C61 butyric acid methylester (PCBM) coated on glass:ITO/ZnO photoelectrode (Figure 10d).^83^ In this architecture, the InP/ZnO/ZnS QDs were designed to delocalize the excited electrons to the ZnO shell and to confine holes in the InP core. Furthermore, subsequent ZnO and ZnS shells were grown on the InP core and increased the photoluminescence quantum yield from 7% (only core) to 28 and 70%, respectively, indicating the passivation of nonradiative surface defects.^150^ The electronic properties of the proposed QD were investigated by quantum mechanical calculations^151^ and compared with InP/ZnS QD. For InP/ZnS QD, the electron and hole are confine to the InP core but a fraction of the electron wave function penetrates to the ZnS shell, indicating smaller exciton binding energy for the shell in comparison with a single InP core QD. However, in InP/ZnO/ZnS QDs, the electron density is not fully confined in the ZnO shell but instead expands through the entire nanostructure, whereas the hole is fully confined in the InP core. This behavior can be attributed to the smaller effective mass, smaller spatial volume, and potential depth.^83^ The decrease in the attractive Coulomb energy, i.e., the binding energy, due to spatially spread electron density and electron delocalization to the shell reveals the formation of the type II heterostructure,^69^ which was also apparent in the electron–hole wave function overlap ratios of 0.89, 0.76, and 0.52 for the InP core, InP/ZnS, and InP/ZnO/ZnS QDs, respectively.

The InP/ZnO/ZnS QD was used as the donor and PCBM as the acceptor, and the blend showed rapid charging/discharging phases with quick rise/fall times of 200 μs. Moreover, the blending ratio, i.e., the number of acceptors per donor, plays a significant role in the photoresponse in terms of capacitive and faradaic processes. QD:PCBM volume with mixture ratios of 1:1, 1:3, and 1:7 resulted in capacitive/faradaic current ratios of 1.43, 2.5, and 1.47, respectively (Figure 9e, f). Therefore, the efficient charge separation, which is essential for capacitive charge generation, requires a sufficiently high and balanced number of acceptors per donor that is satisfied with a 1:3 blending ratio. The benchmark experiments for this blending ratio revealed photovoltage generation of 46 ± 4 mV under 445 nm, 57 mW cm^–2^ pulsed LED illumination. Comparatively, InP/ZnS based control device (glass:ITO, ZnO, InP/ZnS) showed slower charging/discharging phases with rise/fall times of 2 ms, indicating more resistive pathways for charge generation. The superior performance of InP/ZnO/ZnS-based biointerface over InP/ZnS based one can also be explained by the lower exciton binding energy of InP/ZnO/ZnS QD that increases the efficiency of the charge separation.^152^ Thus, this study shows a novel perspective to combine novel heterostructures with fullerene materials to design nontoxic nanoheterojunctions for neural stimulation, which motivates further QD:fullerene combinations for efficient optoelectronic architectures.

### 4.4 PbS QD based neural interfaces

PbS QDs offer strong absorption from visible up to near-IR spectral range.^153^ Moreover, in conjunction with polymers it can have enhanced nanomorphologies for effective charge dissociation. Because the band energy levels of PbS QDs have been conveniently used for bulk heterojunction (BHJ) solar cells, Srivastava et al.^71^ utilized PbS QDs in a blend of the organic donor of P3HT and acceptor of PCBM for photostimulation of neurons. PbS QDs increased the overall absorbance by 14% and net absorbance by 3% at the pump wavelength of 450 nm in comparison with the control group without QDs. Moreover, the efficiency of the charge separation in the photoactive layer also depends on the phase separation between the domains in the BHJ of the P3HT:PCBM blend. The atomic force microscopy revealed smaller intermixed phase-separated domains for P3HT:PbS QDs:PCBM film with better homogeneity in comparison with the P3HT:PCBM film.^71^ Likewise, the surface roughness of the PbS QDs (1.03 nm) integrated film is higher than the control (0.51 nm), respectively.^71^ This increase in surface roughness may also increase the charge collection between the interfaced layers. The benchmark values for the design indicated higher photocurrent generation performance for the optimized photoactive layer thickness, making the photovoltaic device a good candidate for neural interfaces. Furthermore, using PbS QDs in blend form with P3HT and PCBM may reduce the toxic effects for the cellular environment in comparison with the use of PbS as a single interfacial layer or without blending. The investigation of the cell viability and cell growth of SH-SY5Y cells seeded on the fabricated photoelectrodes revealed nonsignificant differences in cell viability tracked by MTT and tracking assays.^71^ This design also utilizes intermediate layers to properly separate electrons and holes, enabling the charge generation dominated by the capacitive processes (Figure 10a, b). Patch-clamp electrophysiology experiments on the SH-SY5Y model cell line indicated that the biointerface can generate peak depolarization of ∼5 mV under low light intensities (1 mW cm^–2^) (Figure 10b), which also eliminates thermal effects that may induce to the cells. Therefore, this study showed the potential and safe use of PbS QDs mixed with organic photoactive polymers, motivating the use of different QDs and their blend with photoactive polymers for designing neural interfaces with better absorption and charge injection. Moreover, toward low-cost, large-scale, and safer synthesis of PbS QDs, greener precursors (i.e., thioacetamide (TAA)) have been proposed as the sulfur precursor.^154^ Together with the advances in colloidal synthesis of QDs, PbS QD-based biointerfaces may offer unique features with NIR light absorption.

For further optimizations, the biointerface architecture glass:ITO/ZnO/P3HT:PbS QDs:PCBM was investigated. The study unrevealed that the weight percent of the PCBM blended with P3HT becomes a significant factor for optoelectronic performance by altering the device absorption. The adaptation of PbS QDs was further optimized in terms of device responsivity in P3HT:PCBM blends.^101^ For that, different photoactive blend thicknesses (Figure 10c, d) and PbS QD loading ratio were investigated, and the optimum device thickness and loading ratio of 155 nm and 10 vol % were determined, respectively.^101^ The atomic force microscopy revealed smaller intermixed phase-separated domains for a 155 nm thick P3HT:PCBM film with better homogeneity in comparison with the 210 nm thick P3HT:PCBM film (Figure 10c). Although reducing the blend thickness results in smoother surface morphology, increased inhomogeneity reduced the capacitive photocurrent. Therefore, there is an optimum surface roughness and surface homogeneity resulting in the best photocurrent injection performance (Figure 10d). The optimized device can induce 0.61 μC cm^–2^ under a 20 mW cm^–2^ intensity of green light with a high responsivity of 99 mA/W. This charge level is above the required threshold levels for neural stimulation of retinal tissue,^155^ and advantageously, the charge generation process was dominantly capacitive. Moreover, the device was responsive to all visible spectrum, also suggesting the potential use for stimulating the retinal tissue. Electrophysiology experiments in vitro on PHNs extracted from E15-E17 Wistar Albino rats showed that the biointerface may elicit action potentials under 20 mW cm^–2^ illumination with very high duty-cycle pulsed stimulation called burst waveforms. The use of burst waveforms for stimulation enables the biointerface to work under ocular safety limits and fast charge accumulation in the cellular environment causing action potentials. Thus, this study suggests the optimization of nanomorphology can build hybrid material systems combining QDs with organic photoactive polymers for neural stimulation. However, in vivo cytotoxicity of PbS-based neural interfaces should be carefully studied for further studies because of its heavy-metal content.

### 4.5 AlSb QD-Based Neural Interfaces

A new type of colloidal nanocrystals, aluminum antimonide (AlSb), was recently introduced in 2019^156^ and is a less studied member of the III–V semiconductors. Physical growth methods^157^ have been already utilized for the synthesis of AlSb semiconductors, particularly for near-IR optoelectronics^158^ and quantum cascade lasers.^159^ However, the introduced tunable colloidal synthesis of AlSb QDs provides the opportunity for solution-processable fabrication. Although most of the available colloidal quantum dots cover the blue and green windows in terms of absorption, the relatively narrow AlSb absorption spectrum in the blue region with a decaying tail for wavelengths longer than 450 nm can enable blue-light-selective optoelectronic performance. Moreover, a direct bandgap with HOMO and LUMO energies of −4.6 and −2.9 eV, respectively, makes AlSb NCs a perfect candidate for being adopted as a hole transfer layer (HTL) to be used with photoactive polymers (Figure 10e). The energy levels are convenient for integration with organic photoactive polymers such as P3HT, PCBM, ITIC, and PTB7-Th.^138,160,161^ Inspired by classical photovoltaic device design, Han et al. developed the ITO/ZnO/P3HT/AlSb QD biointerface (Figure 10e) for neural stimulation of PHNs.^102^ The band alignment between the compounds enabled convenient dissociation of photogenerated excitons in a photoactive P3HT polymer, routing electrons to the ITO layer and holes toward the AlSb QD layer. This proper alignment and effective dissociation generated an induced electric field gradient to the surrounding environment upon illumination. To prove the contribution of AlSb QDs, we compared the photoresponses under 445 nm blue and 630 nm red LED illumination. As expected from the absorption profile of AlSb QDs, the biointerface showed a nonsignificant performance increase under red light but a 2.3-fold higher photocurrent generation under blue light. Photoelectrochemical characterization^138^ of this biointerface showed sufficient charge levels of 0.19 μC cm^–2^ to stimulate neurons in vitro. Advantageously, photoinduced charge generation showed a highly capacitive process with suppressed faradaic charge injection, only 0.92% of the total charge injection. On the other hand, the photochemical stability of QD-based neural interfaces in an aqueous environment has either never been studied or has not been fully evaluated in previous studies. The passive accelerated aging test with an acceleration factor of 32, conducted for 810 h, showed more than 36 months of operational lifetime in this study.^102^ Biocompatibility tests in vitro showed no apoptosis or significant difference in cell viability for extracted PHNs.^102,162^ The electrophysiology experiments on PHNs grown on the biointerface revealed efficient neural stimulation (Figure 10f, g) under 445 nm LED illumination up to 20 Hz (Figure 10g) with low jitter and latency. Advantageously, neural stimulation via the toxic-heavy-metal-free QDs is based on capacitive processes under low light intensities as low as 10 mW cm^–2^ under ocular safety limits.^102,117^ Therefore, it is beneficial to combine different QD systems with photovoltaic devices either as the photoactive material or the interfacial layer as the ETL/HTL. There is still room for enhancement in device efficiency and responsivity, and getting inspiration from the methodologies in solar cell research to increase the device performance could result in superior architectures for high-frequency neural stimulation in different optical excitation windows.

### 4.6 Biocompatibility of QDs and Safety of the Stimulation

**Figure 11:**
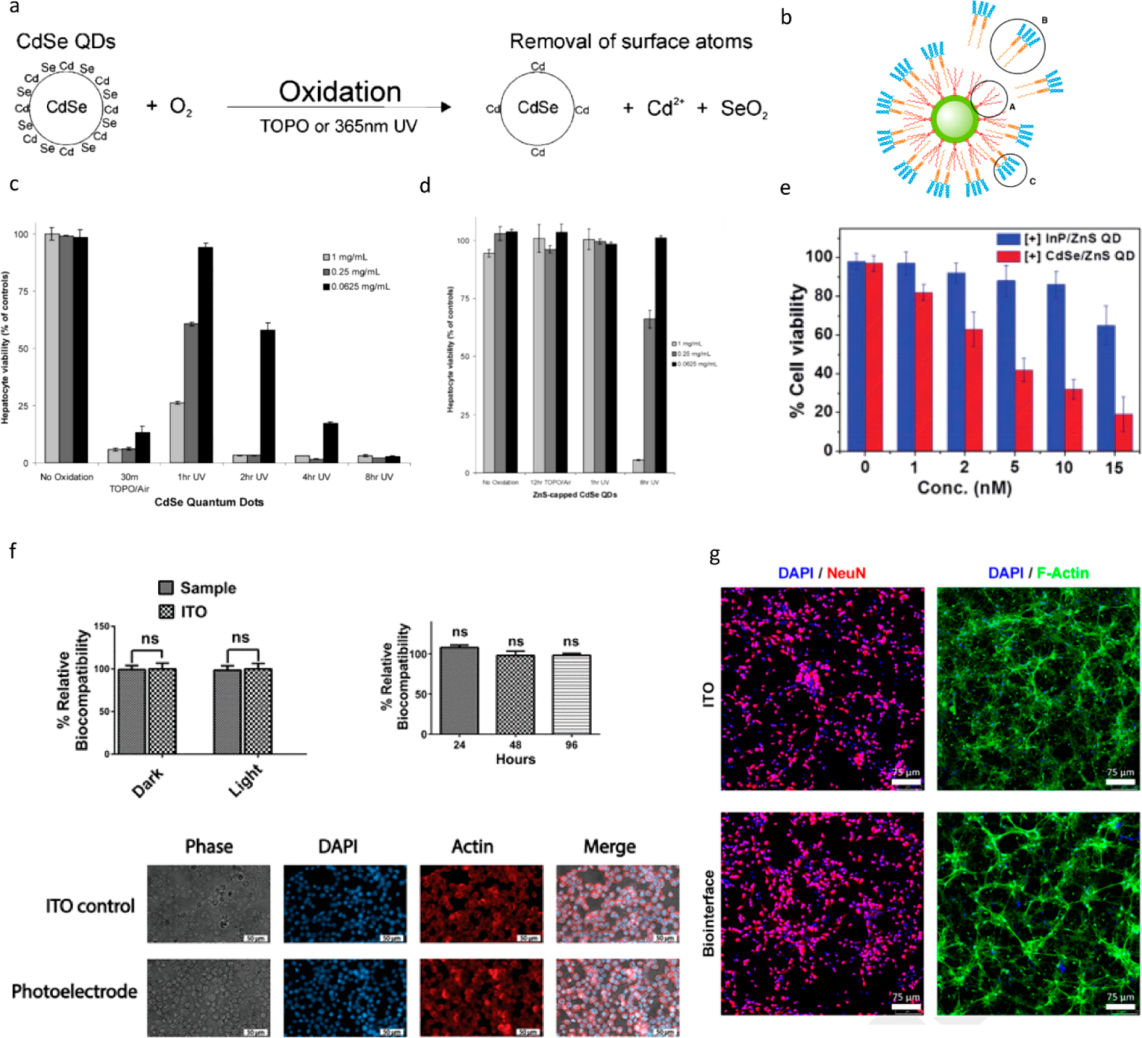
Biocompatibility of quantum dots for biomedical applications. (a) Oxidation mechanism of Cd-based nanoparticles. Reprinted with permission from ref (164). Copyright 2004 American Chemical Society. (b) Polymer encapsulation strategy for colloidal quantum dots. (A) Native nonpolar ligands remain intact and (B) amphiphilic polymer encapsulate the QD for water solubility. (C) Chemically reactive and polar group for bioconjugation. Reprinted with permission from ref (165). Copyright 2011 Elsevier. (c) Cell viability of hepatocytes as assessed by mitochondrial activity of CdSe QD-treated cultures relative to untreated controls under exposure to air and UV treatment. Reprinted with permission from ref (164). Copyright 2004 American Chemical Society. (d) Effect of ZnS coating on CdSe quantum dots on cytotoxicity and oxidation. Reprinted with permission from ref (164). Copyright 2004 American Chemical Society. (e) Cell viability of MCF-7 cells incubated with different concentrations of InP/ZnS QDs and CdSe/ZnS QDs for 24 h. Reprinted with permission from ref (167). Copyright 2017, Royal Society of Chemistry. (f) Cell viability and cytotoxicity assessment of InP/ZnO quantum dots with MTT (upper left), LDH assay (upper right), and visualized cell morphology via DAPI staining and actin immunolabeling (bottom, scale bar: 50 μm). Reprinted with permission from ref (69). Copyright 2018 American Chemical Society. (g) Immunofluorescence imaging of primary hippocampal neurons grown on AlSb NC-coated biointerfaces. PHNs costained with DAPI, Anti-NeuN (red), and anti-F-actin (green) (scale bar: 75 μm). Reprinted with permission from ref (102). Copyright 2021 Springer Nature.

Another key challenge for implantable neural interfaces is to design and fabricate biocompatible devices while maintaining prolonged functional lifetime and efficiency in vivo. The main drawbacks for QD-based devices are (i) ion release from QDs, which might be potentially toxic or change the extracellular pH and reduce device performance, and (ii) the cellular intake by endocytic pathways.^163^ A comprehensive study by Derfus et al.^164^ investigated the cytotoxicity of CdSe QDs and proved that Cd-based QDs can induce toxicity under specific conditions. Particularly, surface oxidation may form Se–O_2_ molecules with desorption of Cd ions (Figure 11a), which inherently induce heavy metal toxicity. As investigated by other studies,^165^ surface coating of QDs with either shells or various inert ligands slows down the surface oxidation processes, improving the biocompatibility (Figure 11b). Various surface coatings were previously used in the literature such as polyacrylate,^110^ bovine serum albumin (BSA),^166^ and ZnS, and they were proved to decrease the surface oxidation process.^164^ Moreover, the role of UV-light excitation on increased cytotoxicity can be attributed to the enhanced oxidative effect of UV-light and elevated levels of free cadmium release from the QDs (Figure 11c).^164^ Although ZnS and BSA surface coatings significantly reduce the cytotoxicity, it is not fully suppressed (Figure 11d). Different QD-based neural interface systems such as InP, PbS, and AlSb QDs were utilized. Particularly, InP QD-based systems showed superior cell viability^167^ due to nontoxic material compounds in the final system (Figure 11e). Moreover, the effect of QDs on cellular processes were evaluated by investigating different biological aspects such as cell metabolic activity (using a 3-(4,5-dimethylth-iazol-2-yl)-2,5-diphenyltetrazolium bromide (MTT) assay) (Figure 8f), cellular cytotoxicity (using a lactate dehydrogenase (LDH) assay to evaluate plasma membrane damage) (Figure 11f), and membrane viability (using immunofluorescent materials such as 4′,6-diamidino-2-phenylindole (DAPI), a marker for membrane viability) (Figure 11g). The readers can refer to the references for more detailed QD cytotoxicity assessments.^20,166,168^ Therefore, it is essential to evaluate the cytotoxicity of QDs for the targeted cells, tissues, and organs, both in vitro and in vivo, as well as under different concentrations, doses, and environmental conditions with properly engineered ligands and shells.

QD-based biointerfaces modulate neural activity by electrochemical currents resulting from conversion of optical energy to electrical energy. The damage mechanisms and charge injection thresholds for electrical stimulation have previously been investigated in detail.^85,169−171^ The QD-based biointerfaces reported up to date have photogenerated charge densities on the order of few μC cm^–2^ and current densities of maximum few mA cm^–2^, which are typically below the damage thresholds for brain and retina.^169,172^ On the other hand, attention must be paid while using the biointerfaces that generate charge-imbalanced monophasic stimulation pulses^88,113^ to avoid possible electrode and tissue damage, although charge-balance does not necessarily indicate electrochemical balance (see Merrill et al. for more on this^85^). Therefore, capacitive biointerfaces are favorable and they typically provide charge-balanced biphasic waveforms.

## 5 Perspective & Conclusion

Quantum-dot-based neural interfaces showed remarkable progress in terms of responsivity and transition toward nontoxic quantum dots. As a next step, seamless integration with targeted tissue via minimally invasive methods while simultaneously increasing spatiotemporal resolution and efficiency remains as an important challenge. For that purpose, the transition toward “single-nanocrystal-level” neural interfaces hold high promise. However, there are fundamental challenges that need to overcome at nanoscale. One of the challenges is the realization of nanocrystals made of metal, oxide, and semiconductor heterojunctions with large lattice mismatch for the control of the photogenerated charges. For example, the metal–semiconductor heterojunctions generally have high lattice mismatch and thus the semiconductors cannot be grown at high crystal quality. As a solution, nonepitaxial growth technique allows the deposition of a crystalline overlayer on a crystalline substrate with high lattice mismatch.^173,174^ For the heterojunctions with low lattice mismatch, epitaxial growth techniques such as a successive ionic layer adsorption and reaction (SILAR) can be applied. Hence, the movement of the photogenerated charge carriers can be well controlled for the targeted charge-transfer mechanism at the electrode–electrolyte interface. Moreover, anisotropic growth of the crystal may lead to spatially separate stimulation and return nanoelectrodes for efficient modulation of neural activity.

After nanocrystals are properly designed and synthesized, they can be conjugated with functional groups (secondary antibodies) and specifically bind to external motifs of neuronal membrane proteins (antigens) via primary antibodies. Once bound to a neuron, these nanocrystals can transduce pulses of light into capacitive or pseudocapacitive currents to modulate the transmembrane potential. The photocurrent can be generated via photogenerated potential change between the nanocrystal and cellular environment that can stimulate or inhibit the neuronal activity at single-cell level. Thus, the combination of single-nanocrystal-level neural interfaces with targeted delivery to the nervous system can be beneficial to treat a wide variety of nervous system diseases at an unprecedented degree. However, state of the art QD-based systems require short-wavelength excitation, which limit their effective use in in vivo therapeutic applications because of the short penetration length of visible light into targeted tissues. Upconversion nanoparticles (UCNPs) and their hybrid systems with QDs enable the use of low-energy NIR light for photostimulation of neurons to overcome this challenge. UCNPs absorb low energy photons, convert them to high-energy visible photons, and excite QDs.^175^ UCNPs have attracted tremendous interest in optogenetics, bioimaging, and light-activated drug release.^175^ Numerous studies in these areas enabled efficient and biocompatible UCNP systems. Particularly, their efficient use in neural modulation has been investigated as the visible photon sources required by optogenetics.^176−180^ Moreover, plasmonic nanostructures have been utilized to enhance upconversion efficiency^181,182^ and their stand-alone use was also studied for modulation of neural activity.^183,184^ Therefore, hybrid systems of UCNPs, photoactive polymers, and plasmonic nanostructures with QDs can offer numerous advantages and opportunities for wireless in vivo studies and clinical research.

Although QDs in free-standing conditions have not yet achieved modulation of neural activity, it has been shown that free-standing silicon nanowires can reproducibly evoke action potentials in primary neurons via the photoelectrochemical pathway.^142^ The ability to build p-type/intrinsic/n-type (PIN) silicon nanowires and to control the doping profiles in a sensitive way render these nanostructures versatile candidates for opto-bioelectronic applications.^185,186^ Moreover, they can also be integrated in composite mesh structures for building flexible and conformable biointerfaces.^185^ Up to now, these systems have operated with high optical power densities using lasers compared to the QD-based systems, which were mostly excited with the light-emitting diodes operating under lower irradiance levels. On the other hand, the ability to modulate neural activity via a single free-standing nanowire motivates the use of single-QD systems for achieving spatially selective stimulation instead of using planar solid films of QDs. With these future improvements, quantum opto-bioelectronics can advance optical control of the nervous system, broaden operational spectral windows, and improve functionality.

On the other hand, more extensive and thorough evaluation of biocompatibility and acute/chronic immune response are needed, particularly for colloidal use of QDs. QD-based biointerfaces can be either injected like nanoparticle-based systems or implanted as building blocks of biointerfaces to the targeted tissue.^29,30^ To date, various techniques such as surface coatings with biocompatible materials such as silica, which is used in various optical^187^ and biomedical applications,^188^ BSA, ZnS, and ZnO, which are dietary molecules,^164^ have been used for reduced cytotoxicity. However, they may either reduce device efficiency or are not sufficient to fully eliminate long-term biological effects. Collaborative efforts of nanoengineering, material science, and bioelectronics can offer new material opportunities to simultaneously achieve efficiency and biocompatibility to address this challenge.

Though neural stimulation is mostly used for direct activation/inhibition of neural activity, there is an increasing and extensive research on its potential in neural differentiation and regeneration.^189^ Although electrical stimulation is the most well-studied and established technique on this subject, recent studies^42,189,190^ showed that optoelectronic stimulation via nanomaterials,^191−193^ particularly nanoparticles,^29,194^ can bring new advantages as an alternative to electrical stimulation with the progress in cellular-scale optoelectronics.^195^ In addition, there are alternative stimulation methods such as magnetic, ultrasound, and a combination of the multimodal approaches that can lead to unconventional neurostimulation strategies.

Monitoring action potentials in neurons via electric-field-modulated QD photoluminescence is a recently developed technique for recording purposes.^22^ Conventionally, the optical readout of neural activity is achieved by chemical Ca^2+^ indicators. However, the photoluminescence kinetics of commercial Ca^2+^ indicators are much slower than the neural activity time scale (e.g., 10–100 s for indicators vs. 1–100 ms for neural voltage signals).^22^ Comparatively, QDs can operate at recombination lifetimes with a resolution of tens of nanoseconds range. The electric field within the vicinity of the cell membrane can couple to the QDs that can shift emission intensity, photoluminescence peak, and emission bandwidth.^196−200^ Furthermore, Förster resonant energy transfer (FRET) between the QD–quencher pair^200^ can be also used for imaging of neuronal action potentials, which can be measured and interpreted with conventional spectroscopic devices. In addition, near-unity QDs can be either used for efficient FRET-systems or integrated into photovoltaic device architecture where the nontransferred or dissociated remaining excitons can efficiently recombine and the luminescence signal may be used for sensing in different configurations. Because QDs with a higher quantum efficiency yielded higher photocurrents due to nontrapped charges, near-unity QDs can be an interesting candidate for simultaneous sensing and stimulation devices.^69,88^ Moreover, such devices can be a powerful alternative to change metabolic activity and monitor drug-induced effects for pharmalogical profiling, in addition to the recent label-free techniques.^185,201−203^

In summary, we discussed the foundations and progress of colloidal quantum dot based neural interfaces for photostimulation of neurons. Despite recent advances, integration of colloidal quantum dots cannot completely reach the silicon- or polymer-based neural interfaces in terms of device efficiency yet. However, we expect that advances in chemical synthesis techniques and colloidal nanosystems combined with bioelectronics can lead to various alternative QD-based devices for neuroscience and treatment of dysfunctional neuronal circuits. For that, recent advances in the related fields have made a great scientific basis for next-generation noninvasive, ultrasmall, and effective neural interfaces. We hope that the recent efforts and challenges discussed in this review will provide insight for future research and motivation for next-generation scientists on these emerging nanomaterial systems and their use in neural interfaces.
